# Using metabolic abnormalities of carriers in the neonatal period to evaluate the pathogenicity of variants of uncertain significance in methylmalonic acidemia

**DOI:** 10.3389/fgene.2024.1403913

**Published:** 2024-07-15

**Authors:** Dongfan Xiao, Congcong Shi, Yinchun Zhang, Sitao Li, Yuhao Ye, Guilong Yuan, Taohan Miu, Haiyan Ma, Shiguang Diao, Chaoyun Su, Zhitao Li, Haiyan Li, Guiying Zhuang, Yuanli Wang, Feiyan Lu, Xia Gu, Wei Zhou, Xin Xiao, Weiben Huang, Tao Wei, Hu Hao

**Affiliations:** ^1^ Department of Pediatrics, The Sixth Affiliated Hospital, Sun Yat Sen University, Guangzhou, China; ^2^ Inborn Errors of Metabolism Laboratory, The Sixth Affiliated Hospital, Sun Yat Sen University, Guangzhou, China; ^3^ Biomedical Innovation Center, The Sixth Affiliated Hospital, Sun Yat-sen University, Guangzhou, China; ^4^ Department of Bioengineering, College of Food Science, South China Agricultural University, Guangzhou, China; ^5^ Neonates Department, Nanhai Maternity and Child Healthcare Hospital of Foshan, Foshan, China; ^6^ Neonatology Departmen, Heyuan Women and Children’s Hospital and Health Institute, Heyuan, China; ^7^ Department of Neonatology, Zhuhai Women and Children’s Hospital, Zhuhai, China; ^8^ Department of Neonatology, Yuebei People’s Hospital, Shaoguan, China; ^9^ Department of Neonatology, Maoming Huazhou People’s Hospital, Huazhou, China; ^10^ Guangzhou Baiyun District Maternal and Child Health Hospital, Guangzhou, China; ^11^ Department of Pediatrics, Huidong County Maternal and Child Health Hospital, Huidong, China; ^12^ Department of Neonatology, The Maternal and Child Healthcare Hospital of Huadu, Guangzhou, China; ^13^ Precision Medicine Laboratory, The First People’s Hospital of Qinzhou, Qinzhou, China; ^14^ Huizhou Huiyang District Maternal and Child Health Hospital, Huizhou, China; ^15^ Department of Neonatology, Guangzhou Women and Children’s Medical Center, Guangzhou Medical University, Guangzhou, China; ^16^ Department of Neonatology, The Fifth Affiliated Hospital of Southern Medical University, Guangzhou, China

**Keywords:** methylmalonic acidemia, variants of uncertain significant, mass spectrometry, neonatal period, silico analysis

## Abstract

**Objective:**

To accurately verify the pathogenicity of variants of uncertain significance (VUS) in *MUT* and *MMACHC* genes through mass spectrometry and *silico* analysis.

**Methods:**

This multicenter retrospective study included 35 participating units (ClinicalTrials.gov ID: NCT06183138). A total of 3,071 newborns (within 7 days of birth) were sorted into carrying pathogenic/likely pathogenic (P/LP) variants and carrying VUS, non-variant groups. Differences in metabolites among the groups were calculated using statistical analyses. Changes in conservatism, free energy, and interaction force of *MMUT* and *MMACHC* variants were analyzed using *silico* analysis.

**Results:**

The percentage of those carrying VUS cases was 68.15% (659/967). In the *MMUT* gene variant, we found that C3, C3/C2, and C3/C0 levels in those carrying the P/LP variant group were higher than those in the non-variant group (*p* < 0.000). The conservative scores of those carrying the P/LP variant group were >7. C3, C3/C0, and C3/C2 values of newborns carrying VUS (c.1159A>C and c.1286A>G) were significantly higher than those of the non-variant group and the remaining VUS newborns (*p* < 0.005). The conservative scores of c.1159A>C and c.1286A>G calculated by ConSurf analysis were 9 and 7, respectively. Unfortunately, three MMA patients with c.1159A>C died during the neonatal period; their C3, C3/C0, C3/C2, and MMA levels were significantly higher than those of the controls.

**Conclusion:**

Common variants of methylmalonic acidemia in the study population were categorized as VUS. In the neonatal period, the metabolic biomarkers of those carrying the P/LP variant group of the *MUT* gene were significantly higher than those in the non-variant group. If the metabolic biomarkers of those carrying VUS are also significantly increased, combined with *silico* analysis the VUS may be elevated to a likely pathogenic variant. The results also suggest that mass spectrometry and *silico* analysis may be feasible screening methods for verifying the pathogenicity of VUS in other inherited metabolic diseases.

## 1 Introduction

Methylmalonic acidemia (MMA) is a serious, fatal, and multisystem damage of the most common organic acidemia (Chen et al., 2023). With neonatal screening, the pooled prevalence of MMA increased approximately 3.5 times between 2011 and 2020 (Jin et al., 2022). In China’s Shandong province, Henan province, and eastern areas, the incidence of live births due to MMA is 1:3,920, 1:4,714, and 1:5,590, respectively (Han et al., 2016; Wang, 2019; Yang et al., 2020). More significantly, early identification has improved the prognosis of MMA, and the mortality rate has decreased from 60%–80% to approximately 40% (Fabie et al., 2019; Zhou et al., 2019). Therefore, accurate and rapid diagnosis of MMA may be a key research topic in the field of inherited metabolic diseases (IMD).

MMA is a single-gene autosomal recessive disease; Zhou et al. (2018) reported that approximately 30% of Chinese patients have isolated MMA, and 70% have MMA with homocystinuria. MMA currently includes more than ten related genes and has high genetic heterogeneity. Among these, the variants in *MUT* and *MMACHC* are the most common in China (Zhou et al., 2018; Kang et al., 2020). According to the typical variant evidence types (such as population database, computational data, and functional data), ACMG classifies these variants as “pathogenic”, “likely pathogenic”, “uncertain significance”, “likely benign”, or “benign” (Richards et al., 2015). With the rapid development of gene sequencing techniques, several variants have been identified. Surprisingly, we screened the genes of 1773 newborns in Guangdong Province in 2021 and found that among the MMA-related variant sites, variants of uncertain significance (VUS) accounted for 85% of the variant sites, and approximately 78% were high-frequency variant sites. However, rapid and accurate analysis of the clinical effects of variants other than nonsense and frameshift variants has always been difficult in gene function analyses (Frederiksen et al., 2021). This is particularly true for inherited metabolic diseases requiring early diagnosis and treatment. Alternatively, VUS may cause serious adverse effects. Our research team has previously shown that one patient with homozygous VUS (c.1159A>C) and two patients with compound heterozygous (c.1159A>C and c.1280G>A, c.1159A>C, and c.1106G>A) variants in *MMUT* died because of severe metabolic disorders in the neonatal period, which is why the c.1159A>C variant was classified as likely pathogenic in the ClinVar database (Hui et al., 2019). However, owing to the limited number of cases, the pathogenicity of the c.1159A>C variant and other VUS in *MMUT* and *MMACHC* is worth further verification.

Currently, genetic screening, such as case reports, determination of protease activity *in vitro* fibroblast cultures, and determination of metabolite content using tandem mass spectrometry, is one of the most common methods for identifying the pathogenicity of variants (Kolhouse et al., 1981; Jansen and Ledley, 1990; Wang et al., 2022). However, this approach is often limited by the small sample sizes and complex operating technologies. Previous studies have found that enzyme activity in some hereditary metabolic disease carriers fluctuates between 25% and 52%, and the corresponding metabolites increase accordingly (Tucci et al., 2021). Therefore, interpreting the relationship between variants and the functions of MMUT or MMACHC variant sites using carriers with a large population base may be a new research strategy. In addition, artificial intelligence applications in the medical field have become a popular topic in modern science and technology. The accuracy of natural language processing (NLP) algorithms for the auxiliary diagnosis of rare diseases has reached 88%–98% (Hens et al., 2022). Artificial intelligence can be used as an effective and accurate tool to promote rapid diagnosis and early intervention in rare diseases. Therefore, our research team planned to take the lead in gene sequencing, protein structure analysis, and tandem mass spectrometry data (within 7 days of birth) to evaluate the pathogenic efficacy of VUS in *MMUT* or *MMACHC* gene carriers from 35 participating units in China and establish an MMA-related high-risk database to quickly identify and improve the accuracy and efficiency of early screening for MMA patients.

## 2 Materials and methods

### 2.1 Subject selection and study protocol

**FIGURE 1:**
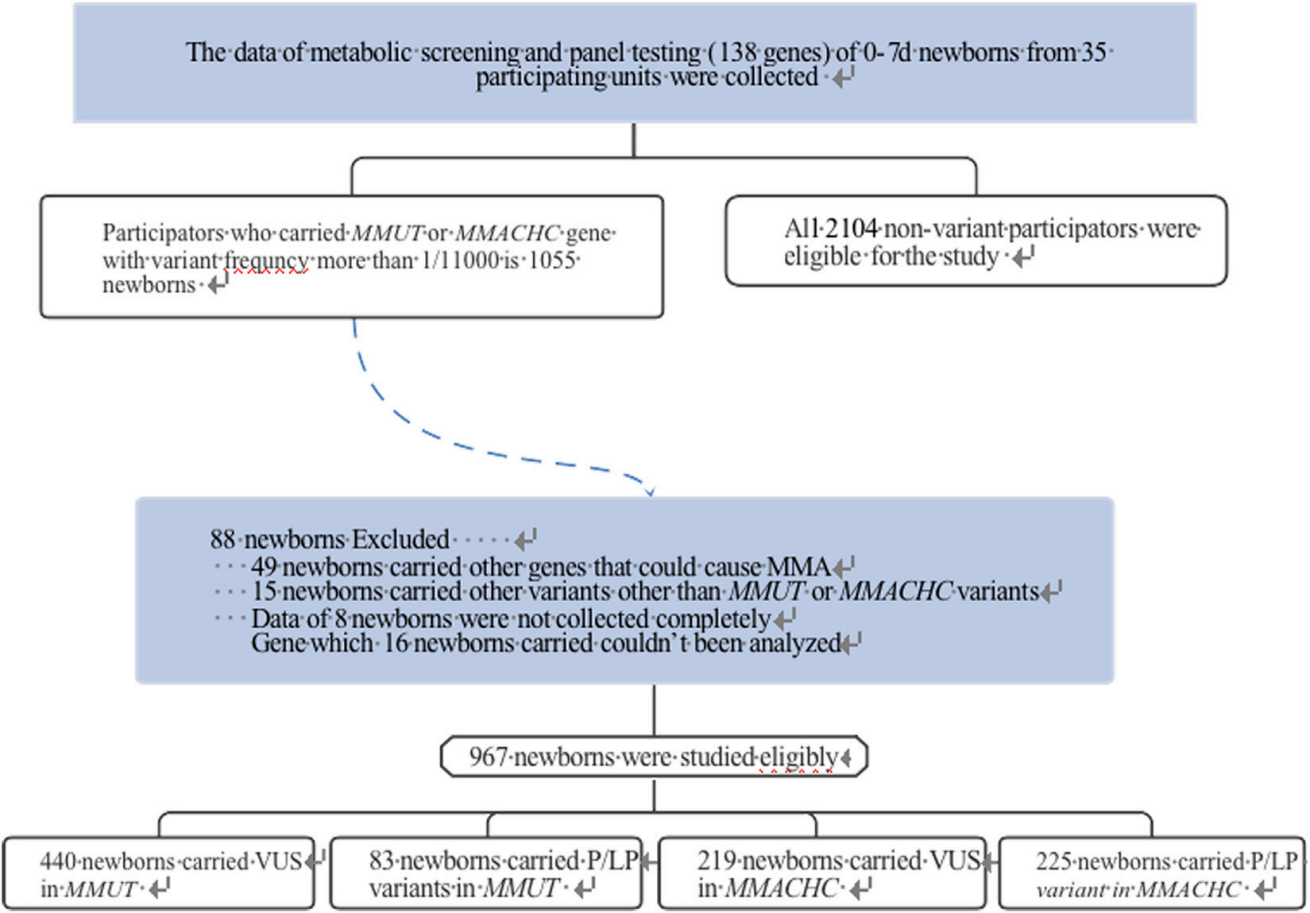
Flow diagram of VUS variant gene research strategy.

Based on gene sequencing panel data including 138 genes associated with 133 common genetic diseases (Supplementary Table S1) and tandem mass spectrometry metabolic group data, 1,055 newborns carrying the *MMUT* or *MMACHC* gene with >1/11,000 variant frequency and 2,104 newborns without genetic variants were selected from 35 participating units in China. Newborns with incomplete data, those carrying multiple variant sites, and other variants in genes (such as *ACSF3*, *MMAA*, *MMAB*, *MMACHC*, *MMADHC*, *LMBRD1*, *ABCD4*, and *HCFC1*) besides *MMACHC* or *MMUT* were excluded (Figure 1). Serum levels of propionylcarnitine (C3), free carnitine (C0), propionylcarnitine/acetylcarnitine (C3/C2), and propionylcarnitine/free carnitine (C3/C0) levels were measured.

### 2.2 Molecular, genetic, and metabolic examination

Three drops of heel blood were soaked in a professional filter paper and dried naturally for gene sequencing and tandem mass spectrometry. A hole punch was used to make a round filter paper of 5 mm-diameter. Genomic DNA was extracted using a magnetic bead blood card genomic DNA extraction kit (BSDE-5005; Shenzhen Uni-Medica Technology Co., Ltd.) and a magnetic bead nucleic acid extraction instrument (NFAST32). The target gene sequence was captured using multiple PCR amplifications (covering all exon regions and adjacent ±50 bp intron regions), and the target sequence library was obtained. The library was quantified using a Qubit 3.0 fluorescence quantitative analyzer (Thermo Fisher Scientific) and its length was determined using an Agilent 2,100 bioanalyzer (Agilent). The library was sequenced using the Illumina NextSeq 500 × 550 platform (PE150). Compared to the human reference genome (Human_B37), variant information was analyzed using the Genome Analysis ToolKit (GATK) and variants were annotated (such as population frequency, variation type, and HGMD/Clinvar) using ANNOVAR software. Variations in pathogenicity were interpreted based on the classification criteria and guidelines for genetic variation (Richards et al., 2015).

The samples for metabolite detection were sent to the Inborn Errors Department of the Metabolic Laboratory of the Sixth Affiliated Hospital of Sun Yat-sen University for LC-MS/MS. Several amino, acycarnitine, and total Hcy determination kits (Zhipu Biotechnology Corporation, Shandong, China) were used for the analysis. LC-MS/MS (Xevo-TQ-DIVD, Waters Corporation, Milford, MA, USA) was used to detect metabolites (Xiong et al., 2019). Metabolite concentrations were automatically calculated using Chemoview software.

### 2.3 Prediction of the functional effect and stability change of amino acid substitution

Wild-type *MMUT*/*MMACHC* FASTA sequences were retrieved from the UniProt website (https://www.UniProt.org/; *MMUT* ID: P22033; *MMACHC* ID: Q9Y4U1). The associated protein sequences were retrieved using the SnapGene tool. Damaged coding non-synonymous SNPs (nsSNPs) were examined to determine whether the protein in question was functional. The pathogenicity and disease association of the nsSNPs were predicted using PANTHER (Protein Analysis Through Evolutionary Relationship), PolyPhen-2 (Polymorphism Phenotyping v2), SNPs, GO, and Sorting Intolerant from Tolerant (SIFT). I-Mutant 2.0, FATHMM (Functional Analysis through Hidden Markov Models), and DeepDDG predicted how nsSNPs would affect protein stability and activity.

### 2.4 Analyzing conserved regions of *MMUT/MMACHC* protein

The ConSurf server (https://consurf.tau.ac.il/consurf_index.php) was used to identify evolutionarily conserved amino acids in *MMUT/MMACHC*, and multiple sequence alignment (MSA) was performed using ClustalOmega. *MMUT/MMACHC* sequences of different species, including *Homo sapiens*, *Pan trogodytes*, *Pongo abelii*, *Macaca fascicularis*, *Sus scrofa*, *Mus musculus*, and *Gallus gallus*, were studied.

### 2.5 3D modeling of *MMUT/MMACHC* protein variants and evaluating the structural consequences

We used the SWISS-MODEL (https://swissmodel.expasy.org/), I-TASSER (https://seq2fun.dcmb.med.umich.edu/I-TASSER/news.html), trRosetta (https://yanglab.nankai.edu.cn/trRosetta/), and AlphaFold2 (https://github.com/deepmind/alphafold) to predict the protein structure of variants. Wild-type and variant protein structures were compared using TM-align (https://zhanggroup.org/TM-align/), which calculates the template modelling-score (TM-score) and root mean square deviation (RMSD) before superimposing the structures. Finally, AlphaFold2 was used to predict mutant structures, achieving a breakthrough in protein structure based on a deep-learning algorithm.

### 2.6 Visualization of structural effect of protein variants

Variants were visualized and analyzed using the HOPE (Have Your Protein Explained) server (https://www3.cmbi.umcn.nl/hope/input/), which identifies the structural effects of point variants in a protein sequence. PyMOL (https://pymol.org/2/) was used to identify three-dimensional mutant residues. NetSurfP-3.0 (https://services.healthtech.dtu.dk/services/NetSurfP-3.0/) was used to determine differences in the secondary structure of the wild-type and variant proteins. Moreover, we used the CASTp (Computed Atlas of Surface Topography of proteins) server (http://sts.bioe.uic.edu/castp/index.html?2cpk) to identify the differences between native and wild-type protein-binding pockets.

### 2.7 Molecular docking

Wild-type and variant protein structures were visualized and analyzed using PyMOL software. We performed molecular docking using Autodock-Vina1.2 to determine the effect of the deleterious variants on the binding affinity of *MMUT*/*MMACHC*. Before using Vina to predict the binding pocket, we employed the protein-plus server (https://proteins.plus/), a tool for predicting protein-binding sites based on neural networks. The docking pocket was confirmed using the GetBox-PyMOL-Plugin (https://github.com/MengwuXiao/GetBox-PyMOL-Plugin) to maximize the docking box. The protein-docking grid box consisted of five different sizes. After docking, we used the protein–ligand interaction profiler (PLIP, https://plip-tool.biotec.tu-dresden.de/plip-web/plip/index) server and PyMOL graphical software for figure generation.

### 2.8 Diagnostic criteria and collection of patient data

The diagnostic criteria for MMA were as follows. 1) Clinical diagnosis: plasma amino acid and acylcarnitine spectrum analysis showed that C3, C3/C0, and C3/C2 increased, C0 decreased, and urine organic acid analysis showed that methylmalonic acid increased significantly. 2) Gene diagnosis panel sequencing analysis revealed two allelic pathogenic variants.

We retrospectively collected clinical data, including 1) clinical symptoms such as poor feeding, vomiting, and seizures; 2) medical treatment; 3) metabolic profile: urine organic acids, plasma amino acid measurement, and blood acylcarnitine profile; 4) gene detection.

### 2.9 Treatment

Initial therapy for these patients included stopping protein intake, intramuscular injection of cobalamin, intravenous, intravenous fluid therapy with glucose and electrolytes, high-calorie diet with special formula supplementation, and symptomatic treatment.

### 2.10 Statistical analysis

Statistical analyses were performed using SPSS 26.0 version (IBM Corporation, Armonk, NY, USA). Analyses using the Kolmogorov–Smirnov test, Shapiro–Wilk test, histogram, and Q–Q diagram showed that the data were non-normally distributed. Therefore, the values were expressed as medians (quartiles), and an independent sample nonparametric test was performed. *p* < 0.05 was considered statistically significant.

## 3 Result

### 3.1 Variant distribution characteristics and metabolic levels of MMA variant site carriers

**FIGURE 2:**
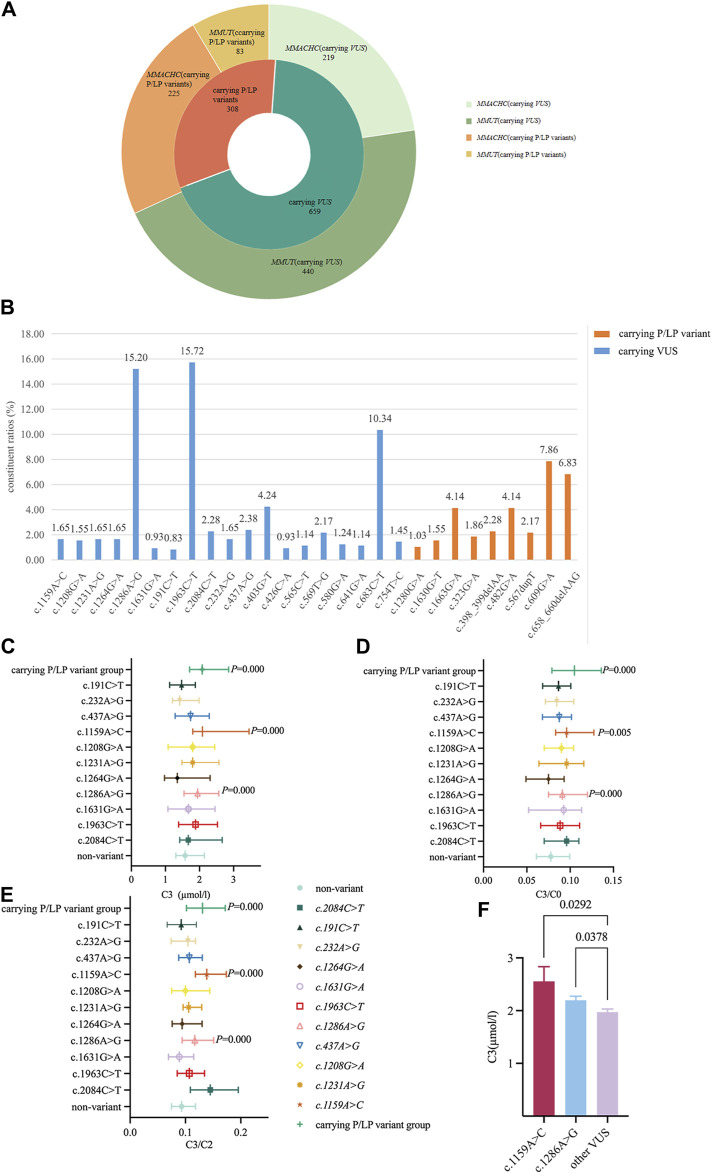
Distribution characteristics and metabolic levels of newborns carrying related variants site. **(A)** Study the distribution of variants (carrier rate≧1/11,000) in *MMUT* and *MMACHC* genes. **(B)** Percentage of variant sites (carrier rate≧1/11,000) in *MMUT* and *MMACHC* genes. **(C)** Changes in C3 levels when carrying 11 VUS in *MMUT.* Data were expressed as medians (P_25_, P_75_). **(D)** Change of C3/C0 ratio when carrying 11 VUS variants in *MMUT*. Data were expressed as medians (P_25_, P_75_). **(E)** Change of C3/C2 ratio when carrying 11 VUS in *MMUT*. Data were expressed as medians (P_25_, P_75_). **(F)** Comparison of C3 levels in newborns carrying the **(C)** 1159A>C or **(C)** 1286A>G variant with other carrying VUS newborns. All data were analyzed using independent sample nonparametric test. Data of Panel C,D,E compared with non-variant group. Data of Panel F compared with other carrying VUS variants except for **(C)** 1159A>C and **(C)** 1286A>G.

The results of neonatal screening from 35 participating units in China showed that 523 and 444 newborns had >1/11,000 carrier rate and only *MMUT* or *MMACHC* variant sites, respectively. The percentage carrying VUS was 68.15% (659/967), which was significantly higher than that for the P/LP variant group (Figure 2A). The constituent ratios of carrying VUS in *MMUT* and *MMACHC* were 84.13% (440/523) and 49.32% (219/444), respectively. The top three variant sites with high constituent ratios were c.1963C>T (15.72%) and c.1286A>G (15.20%) in *MMUT* and c.683C>T (10.75%) of *MMACHC*, all of which were VUS (Figure 2B and Supplementary Table S2).

The C3, C3/C0, and C3/C2 levels of newborns carrying the P/LP variant group were 2.08 (1.711, 2.857) µmol/L, 0.105 (0.079, 0.136), and 0.131 (0.102, 0.172), respectively, which were higher than those of the non-variant group (*p* < 0.000). To further understand the metabolic level changes in the VUS of the MMA gene, we found that C3, C3/C0, and C3/C2 levels of carrying c.1159A>C variant newborns [2.087 (1.803, 3.455) µmol/L, 0.096 (0.083, 0.128), and 0.139 (0.118, 0.174), respectively] as well as carrying c.1286A>G variant newborns [1.944 (1.545, 2.566) µmol/L, 0.091 (0.075, 0.12), and 0.117 (0.094, 0.151), respectively] were significantly higher than those of the non-variant group and the remaining VUS newborns (Figures 2C–F, Supplementary Figure S2). However, no significant changes were observed in the metabolites carrying the *MMACHC* variants (Supplementary Figure S1).

### 3.2 Structural influence and conservation analysis of amino acid sites

Prediction algorithms related to protein structure models have high accuracy for missense variants, and there is no corresponding X-ray wild-type protein structure at base 754 in *MMACHC*; thus, c.398_399delAA, c.567dupT, c.658_660delAAG c.1630G>T in *MUT* and c. 609G>A, c.754T>C in *MMACHC* were not analyzed. *MMUT* and *MMACHC* variants were mapped onto a three-dimensional structure using PyMOL to visualize the effect of these variant residues on the protein structure (Supplementary Figure S3). Wild-type and variant proteins were differentially visualized using the HOPE project (Supplementary Figure S4). The A555T, P695L, G544E, and G427D protein variants were larger than the wild-type proteins, whereas R655C, Y429C, Y146C, T78A, R108H, I411V, and R403Q were smaller than the wild-type proteins and had a different secondary structure (Supplementary Table S3).

The CASTp 3.0 server was used to identify the differences in the binding pockets between the wild-type and variant proteins. The results are summarized in Supplementary Table S4. The area and volume of the wild-type protein were 1.391 and 0.07, respectively, whereas those of the MMACHC V135L variant protein are 2.672 and 0.237, respectively. This is consistent with the findings of HOPE analysis, which showed that V135L had a variant residue larger than the wild-type residue. These variants alter the binding affinities of proteins by changing their binding pockets.

Seven different *in silico* nsSNP prediction tools were used to confirm the function and stability of protein variants. All nsSNPs were evaluated using sequence- and structure-based tools to investigate their deleterious nature. The predicted results are listed in Supplementary Table S5. The conservation level of the residues provides a general estimate of the level of damage caused by deleterious variants. MUT positions 387 and 429 were taller than 64, 78, and 422 (Supplementary Figures S5, S6). ConSurf server results were used to validate the MSA findings. MMACHC position 161 with a score of 9 and the MMUT positions 108, 387, 427, 429, and 555 with scores of 7, 9, 9, 9, and 7 were highly conserved (Table 1 and Supplementary Table S6).

**TABLE 1:** Conservation analysis results predicted by ConSurf for *MMUT* variants.

Gene	Variant sites	Position	Score
*MMUT*	c.191C>T	P64L	3
*MMUT*	c.232A>G	T78A	1
*MMUT*	c.323G>A	R108H	9
*MMUT*	c.437A>G	Y146C	5
*MMUT*	c.1159A>C	T387P	9
*MMUT*	c.1208G>A	R403Q	5
*MMUT*	c.1231A>G	I411V	6
*MMUT*	c.1264G>A	A422T	4
*MMUT*	c.1280G>A	G427D	9
*MMUT*	c.1286A>G	Y429C	7
*MMUT*	c.1631G>A	G544E	1
*MMUT*	c.1663G>A	A555T	7
*MMUT*	c.1963C>T	R655C	2
*MMUT*	c.2084C>T	P695L	4

### 3.3 Molecular docking analysis

The wild-type protein had the lowest binding energy, indicating that it was more stable than the other protein variants. The free energies of R108H, T387P, G427D, and R161Q variant proteins increased significantly after binding to the ligands (−3.259, −4.23, −1.608, and −6.91 kcal/mol, respectively). Compared with that of the wild-type protein, the free energy of P64L, T78A, and A422T variant proteins did not change significantly after binding to the ligand (−0.35, −0.163 and −0.163 kcal/mol, respectively) (Table 2 and Supplementary Table S7).

**TABLE 2:** Docking result of MMUT protein with (*R*)-methylmalonyl-CoA.

Variant sites	Position	Ligand	Wild-type binding affinity (kcal/mol)	Variant binding affinity (kcal/mol)
c.191C>T	P64L	(*R*)-methylmalonyl-CoA	−6.775	−6.425
c.232A>G	T78A	(*R*)-methylmalonyl-CoA	−6.179	−6.016
c.323G>A	R108H	(*R*)-methylmalonyl-CoA	−10.08	−6.821
c.437A>G	Y146C	(*R*)-methylmalonyl-CoA	−6.769	−5.51
c.1159A>C	T387P	(*R*)-methylmalonyl-CoA	−9.221	−4.991
c.1208G>A	R403Q	(*R*)-methylmalonyl-CoA	−6.463	−6.076
c.1231A>G	I411V	(*R*)-methylmalonyl-CoA	−5.863	−5.209
c.1264G>A	A422T	(*R*)-methylmalonyl-CoA	−5.588	−5.425
c.1280G>A	G427D	(*R*)-methylmalonyl-CoA	−6.459	−4.842
c.1286A>G	Y429C	(*R*)-methylmalonyl-CoA	−6.719	−4.775
c.1631G>A	G544E	(*R*)-methylmalonyl-CoA	−5.225	−5.022
c.1663G>A	A555T	(*R*)-methylmalonyl-CoA	−6.842	−3.960
c.1963C>T	R655C	(*R*)-methylmalonyl-CoA	−6.189	−5.738
c.2084C>T	P695L	(*R*)-methylmalonyl-CoA	−5.904	−5.378

### 3.4 Clinical presentation of the case

**FIGURE 3:**
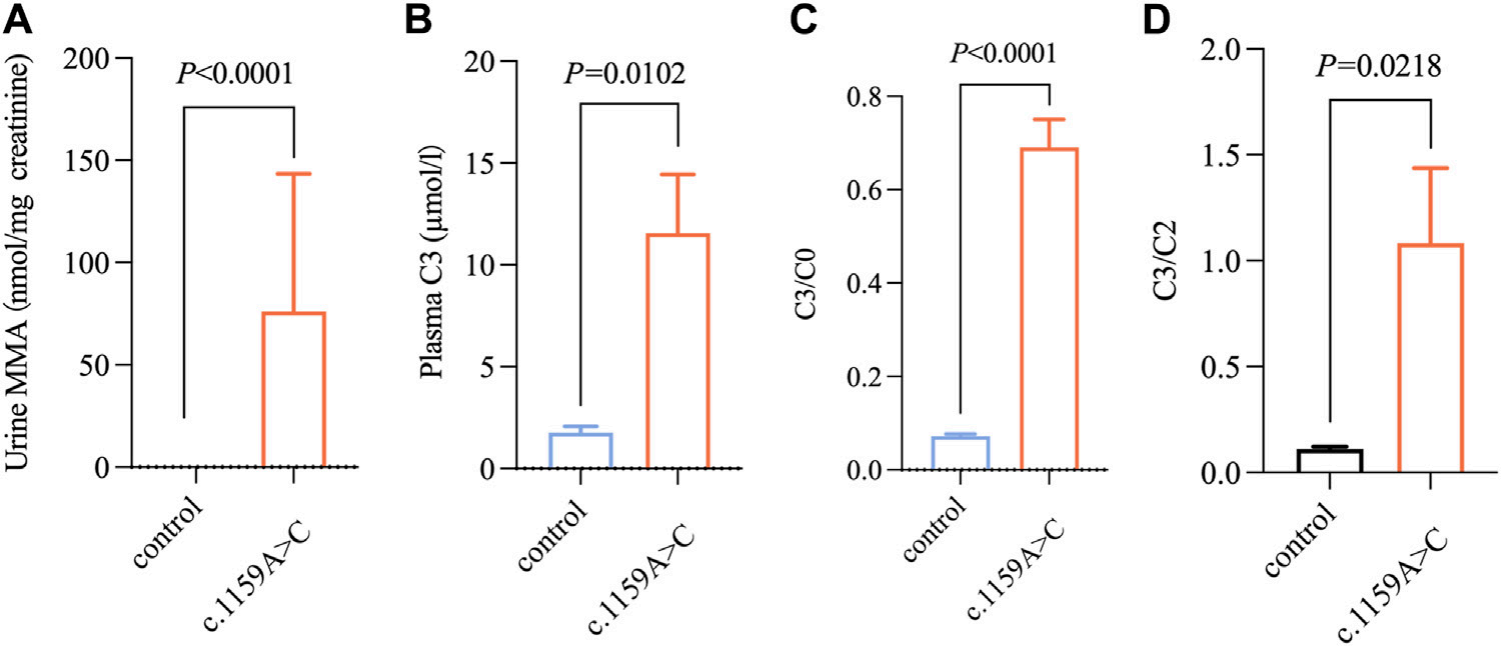
Metabolic level of abovementioned three patients with methylmalonic acidemia. **(A–D)** Changes in MMA, C3, C3/C0, C3/C2 levels in newborns carrying c.1159A>C variation with methylmalonic acidemia (n = 3/group). All the data were tested using the independent sample nonparametric test.

To verify the pathogenicity of the variants, we searched one patient homozygous for c.1159A>C and two patients with compound heterozygosity (c.1159A>C and c.1280G>A, c.1159A>C, and c.1106G>A) from our clinical database. The onset age of all patients ranged 5 d–24 d after birth. The typical initial clinical symptoms included poor feeding, vomiting, acidosis, recurrent vomiting, lethargy, and dyspnea (Table 3). Unfortunately, all patients died of severe metabolic disorders during the neonatal period (Table 3). The plasma C3, C3/C0, and C3/C2 of the three patients were significantly higher than control (*p* < 0.05); the urine MMA levels of those patients were (7.99–210.72) µmol/mg creatinine higher than that in the control group (*p* < 0.001) (Figure 3).

**TABLE 3:** General feature of *c.1159A>C* variation identified from three patients with methylmalonic acidemia.

No	Gene	Nucleotide variant 1	Nucleotide variant 2	Onset ages (d)	Clinical symptoms	Clinical outcome
1	*MMUT*	c.1280G>A	c.1159A>C	6	Poor feeding, vomiting, acidosis	Neonatal death
2	*MMUT*	c.1106G>A	c.1159A>C	24	Poor feeding, dyspnea	Neonatal death
3	*MMUT*	c.1159A>C	c.1159A>C	5	Recurrent vomiting, lethargy	Neonatal death

## 4 Discussion

Our study unveils the cross-sectional data of carrying *MMUT* and *MMACHC* gene variations from 35 participating units in China. Using gene sequencing, protein structure analysis, and metabolic data, our team was the first to reveal the change in the metabolic level of those carrying the P/LP variant group and to evaluate the pathogenicity of VUS, boasting the largest sample size of *MMUT* and *MMACHC* variant carriers reported to date. The new strategy identified the opportunity to reclassify these variants in *MMUT* and *MMACHC* in the current database and may enable us to prioritize and target the pathogenicity of variants in other inherited metabolic diseases for evaluation.

In the NIH database, the VUS of the most common *MMUT* and *MMACHC* genes in MMA account for 38.34% and 34.57%, respectively. We found that 68.15% of the examiners carried VUS by studying the variant of *MMUT* or *MMACHC* gene with only carried and an incidence rate greater than >1/11,000. Among them, the first three variant sites with the highest constituent ratios were all VUS variant; this potential health problem imposes a psychological burden on asymptomatic individuals and affects clinical management. To further classify VUS, the ClinGen expert group adopted a Bayesian framework to facilitate the automatic quantitative evaluation of pathogenicity (Tavtigian et al., 2018). In addition, multidisciplinary cooperation such as clinical phenotypic assessment, in-depth literature research including artificial intelligence, and protein structure analysis have become important tools for VUS classification (Caswell et al., 2022; Casaletto et al., 2023; Farman et al., 2024). However, the pathogenicity of rare diseases still lacks sufficient samples, and artificial intelligence is easily affected by parameter changes. Therefore, accurately evaluating the relationship between variants and pathogenicity or disease clinical phenotypes is a challenge for molecular diagnosis and the implementation of individualized precision therapy.

Liquid chromatography-tandem mass spectrometry (LC-MS/MS) is a highly sensitive and accurate technique for detecting small-molecule metabolites (<1,500 kDa). Since the mid-20th century, it has been widely used in the diagnosis and treatment of genetic and metabolic diseases such as amino acid disorders, organic acidemia, and fatty acid oxidation defects, and has shown considerable effects and prospects in IEM characterization (Vernon, 2015; Piraud et al., 2018; Wang et al., 2019; Zhang et al., 2021). According to the human metabolome database (HMDB), more than 700 metabolites are associated with IEM (Mandal et al., 2018). For example, plasma biomarkers such as oxidized sterols, bile acids, and lysosphingolipids are used to detect sphingolipids and related diseases (such as Niemann-Pick type C) (Piraud et al., 2018). Detection propionylcarnitine using liquid and gas mass spectrometry is the most reliable method for screening prenatal MMA (Chen et al., 2021). Metabolites, as the final functional products of the genome, can be used to analyze genetic variation and explore gene–phenotype correlations. Heterozygous familial hypercholesterolemia with an 1/500 estimated prevalence may lead to metabolic abnormalities (cholesterol, low-density lipoproten, etc) and a 13-fold increased risk of coronary heart disease (Nordestgaard et al., 2013). We identified 83 newborns carrying P/LP variants in *MMUT* and 225 patients carrying P/LP variants in *MMACHC* using genome sequencing. It was revealed the C3, C3/C2, and C3/C0 levels in *MMUT* gene variant carriers were higher than those in the non-variant group, which could be used as an important rationale for exploring the pathogenicity of *MMUT* gene variants. This finding is similar to those observed in other genetic metabolic diseases. Van Acker et al. (1977) found abnormal adenine, 8- hydroxy −9, and 2,8-dihydroxyadenine levels in the urine of six carriers from a male family with complete adenine phosphoribosyltransferase deficiency. Pyrimidine, orotic acid, uridine, and uracil levels also increased in the plasma and urine of heterozygous women with ornithine carbamoyltransferase (OCT) deficiency (Webster et al., 1981) and OTC enzyme activity was 0%–60% of the normal value in a cohort of 281 heterozygous females (Gobin-Limballe et al., 2021). Neonatal screening data were collected. Our research team found that carriers of other metabolic diseases also show metabolic changes such as medium-chain acyl-CoA dehydrogenase deficiency and primary carnitine deficiencies. No significant changes were found in the metabolites carrying *MMACHC* variant sites. It is likely that adequate intake of vitamin B12 during pregnancy and maternal vitamin B12 levels are associated with a better neonatal vitamin B12 status (Aparicio et al., 2020; Li et al., 2021). To analyze the variant types more accurately, we showed that the conservative scores for all three missense variants (c.323G>A, c.1663G>A, and c.1280G>A) were greater than 7. Compared to the wild-type, the free energy of the combination caused by the above three genetic variations was significantly increased. It is speculated that the increased metabolite levels might be related to the activity of mutant methylmalonate coenzyme A transferase (MCM). However, quantitative analysis of enzyme activity and protein structure stability of heterozygous variants in *MMUT* needs further verification in future studies in view of MMA expression intensity in the liver, kidney, and brain of the *Mut* animal model (Forny et al., 2016). Combined with the results of the quantitative analysis of enzyme activity changes in *MMUT* variants, we expect to establish a relative quantitative prediction of metabolites in lesion tissues using a genome metabolism model as the structure in the future.

Based on neonatal metabolic data, artificial intelligence (Random Forest Machine Learning Class Fiber) trained by the Yale University School of Medicine and Stanford Genome Technology reduced the false positive rate of MMA by more than 51%, making it an efficient and cheap auxiliary screening tool (Peng et al., 2019). Therefore, we comprehensively analyzed the metabolic biomarkers, gene sequencing data, and *silico* analysis of 967 *MMUT* and *MMACHC* gene carriers during the neonatal period to predict VUS pathogenicity. The details are as follows. 1) Mass spectrometry data showed that the C3, C3/C0, and C3/C2 levels of newborns with VUS (c.1159A>C and c.1286A>G) were close to those of newborns carrying P/LP variant group and significantly higher than those of newborns with non-variants and the remaining VUS newborns in our study. 2) According to PANTHER, PolyPhen-2, SIFT, FATHMM, and DeepDGG biological tools, these two variants affect protein structure stability and damage biological functions. The conservative scores used to verify the degree of damage caused by VUS (c.1159A>C and c.1286A>G) were 9 and 7, respectively. Molecular docking showed that the free energy of variant ligand binding was significantly higher than that of wild-type ligand binding. In addition, the C3, C3/C0, C3/C2, and MMA levels of three patients with c.1159A>C variation in our clinical database were significantly higher. All the patients died from severe metabolic disorders during the neonatal period. This provides a theoretical basis for the pathogenic properties of c.1159A>C discovered previously by our research group ^[10]^. However, the c.1286A>G variants have not been previously reported, and experimental evidence to identify their biological functions is not available. Nucleotide site-directed mutagenesis screening, which can accurately locate and induce point variants in any genome and identify cell clones with target variants, has been used to explore the functional analysis of Lynch syndrome-related mismatch repair gene variations with 97.6% specificity and 92.3% sensitivity (Houlleberghs et al., 2016). This study provides technical support for further clarification of the pathogenicity of MMA-related VUS, which is beneficial for high-throughput research on its pathogenesis and drug targeting.

## 5 Conclusion

We found for the first time that the C3, C3/C0, and C3/C2 levels in pathogenic/likely pathogenic *MMUT* gene variant carriers were higher than those in non-variant carriers. The c.1159A>C and c.1286A>G variants may be pathogenic based on genomic, tandem mass spectrometry data, and bioinformatics analyses. Therefore, a comprehensive analysis based on genomic, carrier metabolite, and bioinformatics analyses may be a new method for interpreting variant pathogenicity, which is important for early MMA diagnosis and precise treatment and can thus improve patient prognosis and the health of the whole population.

## 6 Limitation

Our study had several limitations. 1) The data were mostly obtained from southern China and may not be representative of the general population. Therefore, we could not establish effective critical values for *MMUT* and *MMACHC* pathogenic variant carrier-related metabolic screening indicators in newborns. 2) We did not verify the physiological status of these carriers after identifying the pathogenicity of the variant site. 3) Considering that *sillico* analysis is primarily designed for missense variants, we were unable to predict the structural and functional outcomes following frameshift and nonsense variants. Moreover, it is difficult for this tool to predict the interactions with partner proteins, multimers, and glycosylation. Consequently, in future research we will add an *in vitro* experiment of primary cells and an *in vivo* experiment of transgenic mouse models to verify the pathogenicity of the variants.

## Data Availability

The data analyzed in this study are subject to the following licenses/restrictions. The data that support the findings of this study are not openly available due to reasons of sensitivity and are available from the corresponding authors upon reasonable request. Requests to access these datasets should be directed to HH, haohu@mail.sysu.edn.cn.
