# Supplementary material for: Using metabolic abnormalities of carriers in the neonatal period to evaluate the pathogenicity of variants of uncertain significance in methylmalonic acidemia
Source: Front Genet. 2024 Jul 15;15:1403913. doi: 10.3389/fgene.2024.1403913 (PMC11284102; doi:10.3389/fgene.2024.1403913)
Supplement: Supplementary file 1 [file Table1.DOCX]

Supplementary Material

# Supplementary Figures and Tables

**Figure S1**. Metabolic levels of newborns with variant sites in the *MMACHC* gene

**Figure S2**. Comparison of C3/C0 and C3/C2 levels in newborns carrying c.1159A>C or c.1286A>G variant with other carrying VUS newborns

**Figure S3**. The wild-type structure of MMUT (PDB code: 2XIJ)and MMACHC*.* (PDB code: 3SBZ). (A) Location of *MMUT* missense variants in red considered in this study. Domain 1 in blue is the Methylmalonyl-CoA mutase (resi 63-574) and domain 2 in yellow is the B12-binding domain (resi 614-764). (B) Location of *MMACHC* misenese variants in red consideres in this study. Domain in blue is the Methylmalonic aciduria and homocystinuria type C family (resi 20-234)

**Figure S4**. Visualization of wild-type amino acid residues and mutated amino acide residues

**Figure S5**. Conservation analysis results of *MMUT* (A) and *MMACHC* (B) as predicted by the ConSurf server

**Figure S6**. The result of MSA

**Table S1.** A list of 138 related genes in the study

**Table S2.** Evidence of ACMG pathogenicity classification for VUS sites in the study in the study

**Table S3**. Structural effects on *MMUT/MMACHC* due to variants obtained from the HOPE Server.

**Table S4**. Differences in the binding pockets of the wild-type protein and mutant proteins identified using the CASTp 3.0 server

## **Table S5**. Predictions for all missense nsSNPs by seven tools with their respective scores

## Table S6. Conservation analysis results predicted by Consurf for *MMACHC* variant sites

Table S7**.** Docking result of *MMACHC* protein with cob(II)alamin

Table S8**.** Number of newborns with carrying variants in *MMUT* and *MMACHC*

**
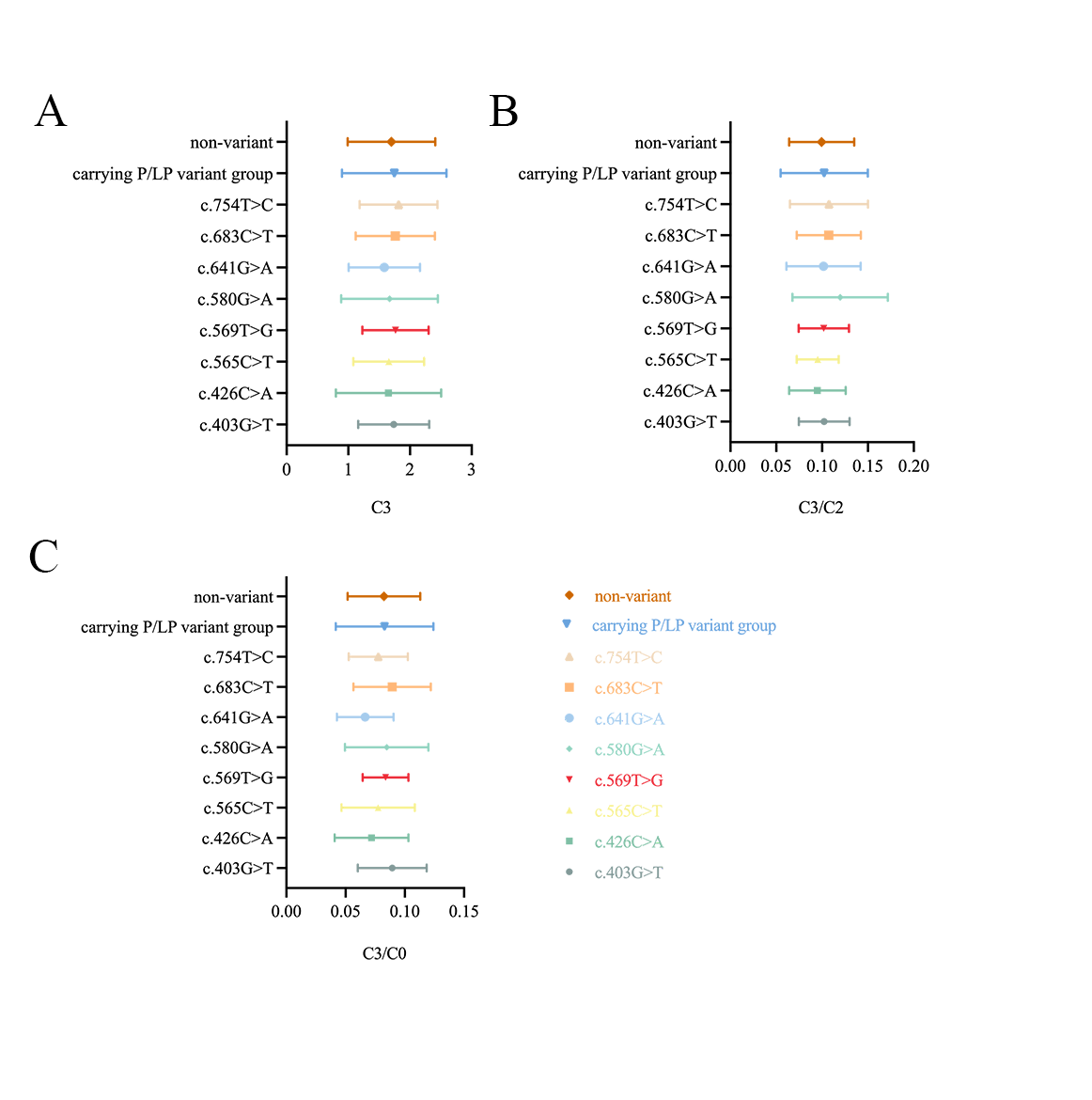
**

Figure S1. Metabolic levels of newborns with variant sites in the *MMACHC* gene.


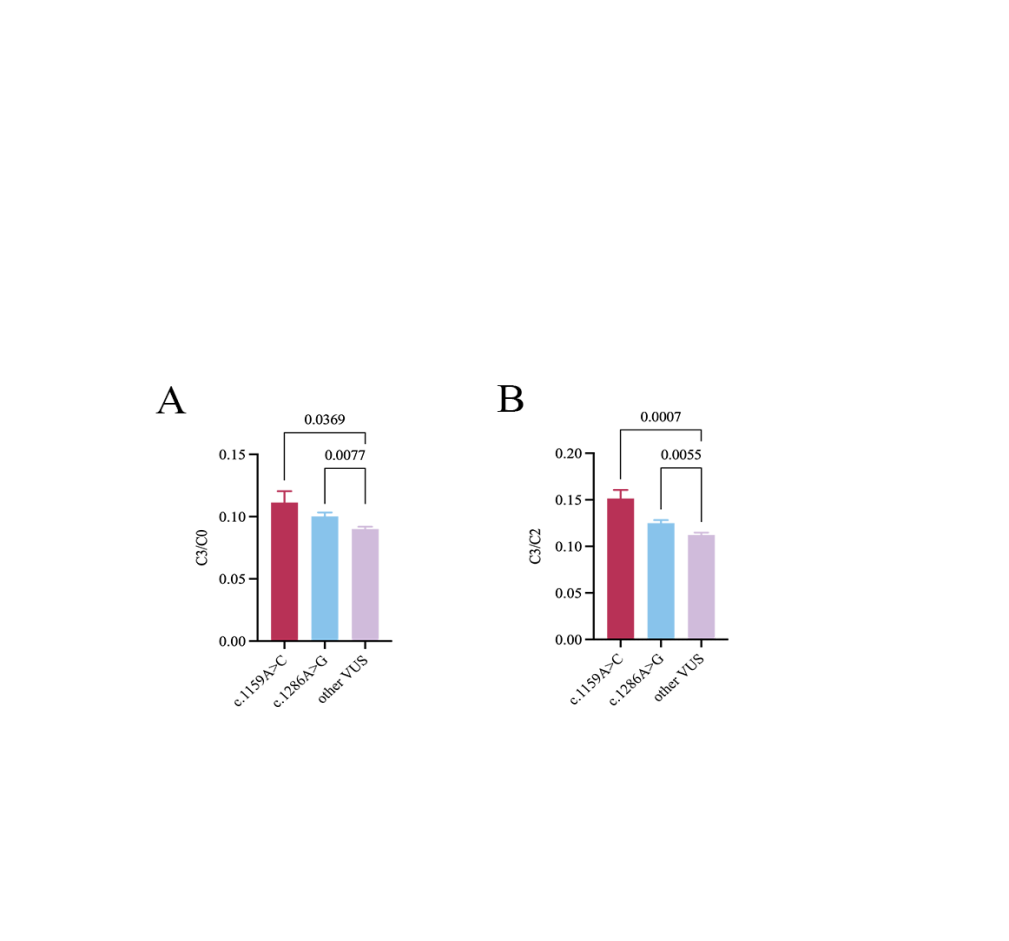


Figure S2. Comparison of C3/C0 and C3/C2 levels in newborns carrying c.1159A>C or c.1286A>G variant with other carrying VUS newborns. All the data were tested by independent sample nonparametric test. Data of figure S2 Compared with other carrying VUS mutation group except c.1159A>C and c.1286A>G.


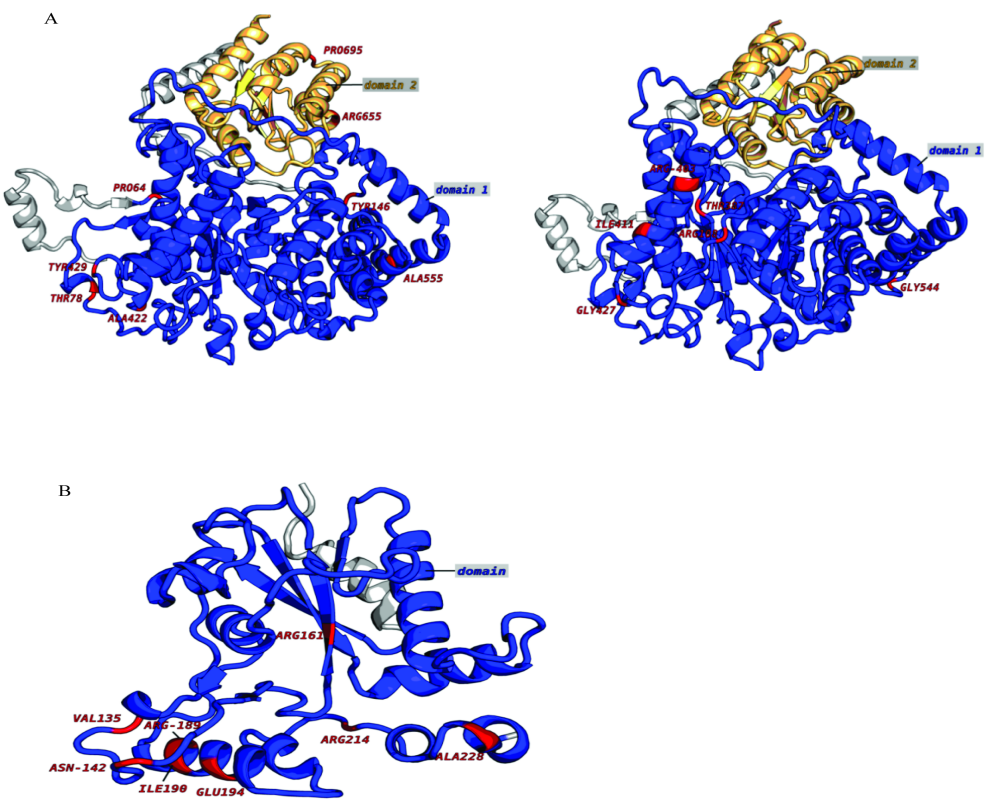


Figure S3. The wild-type structure of MMUT (PDB code: 2XIJ) and MMACHC (PDB code : 3SBZ). (A) Location of *MMUT* missense variants in red considered in this study. Domain 1 in blue is the Methylmalonyl-CoA mutase (resi 63-574) and domain 2 in yellow is the B12-binding domain (resi 614-764).(B) Location of *MMACHC* misenese variants in red consideres in this study. Domain in blue is the Methylmalonic aciduria and homocystinuria type C family (resi 20-234).


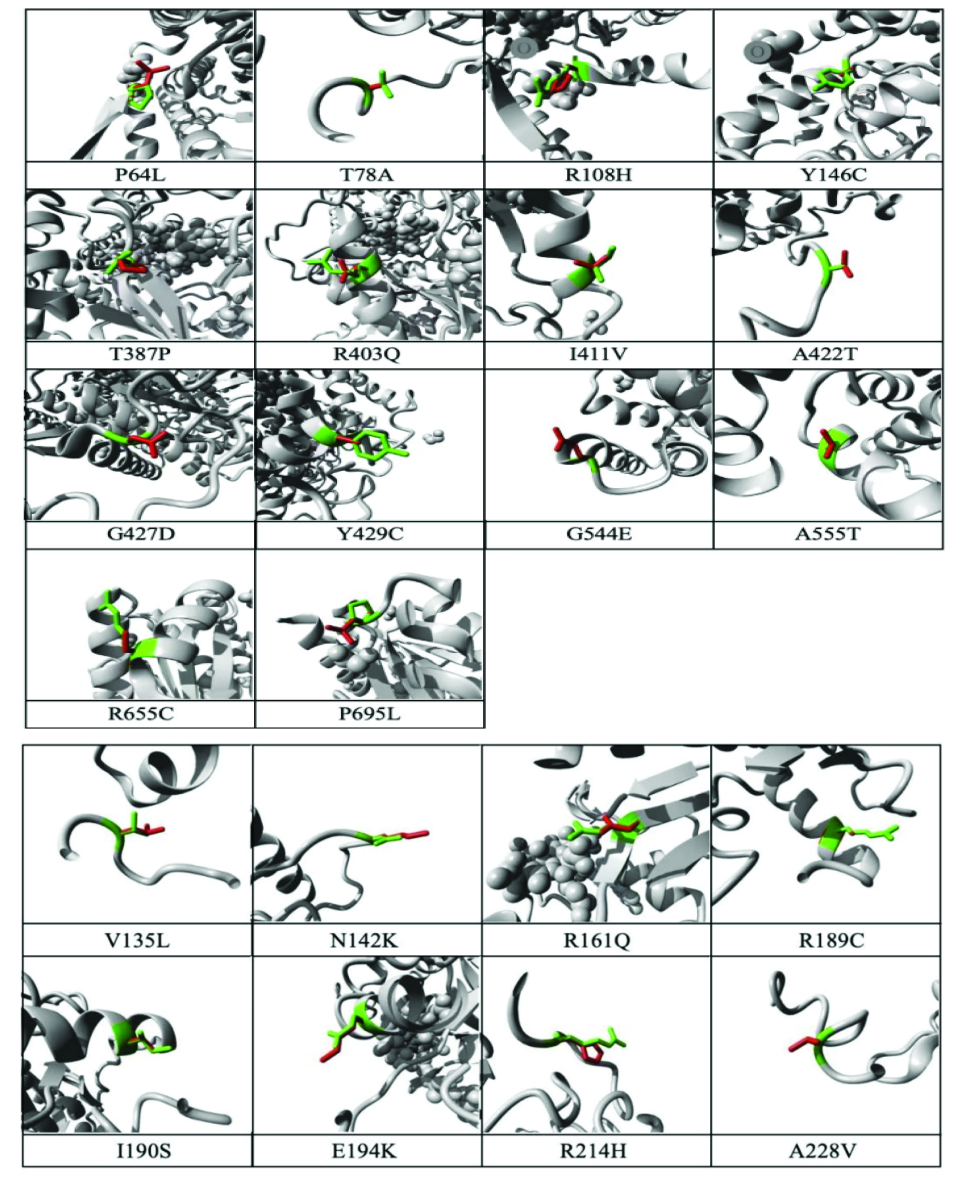


Figure S4. Visualization of wild-type amino acid residues and mutated amino acide residues.


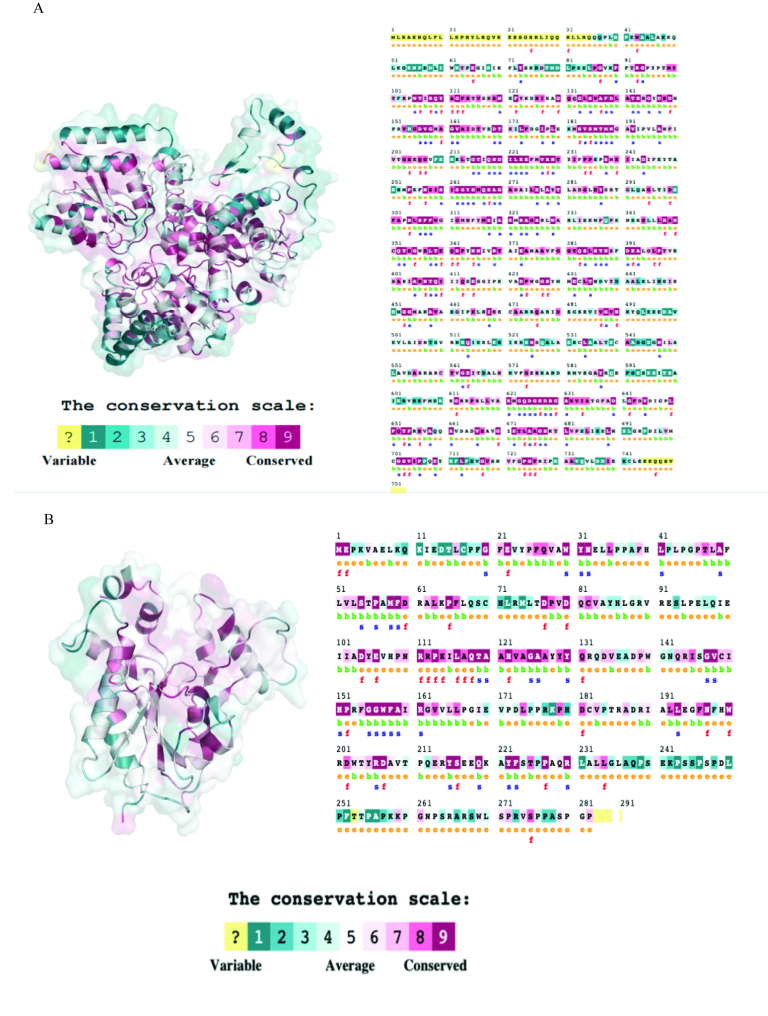


Figure S5. Conservation analysis results of *MMUT* (A)and *MMACHC* (B) as predicted by the ConSurf server.


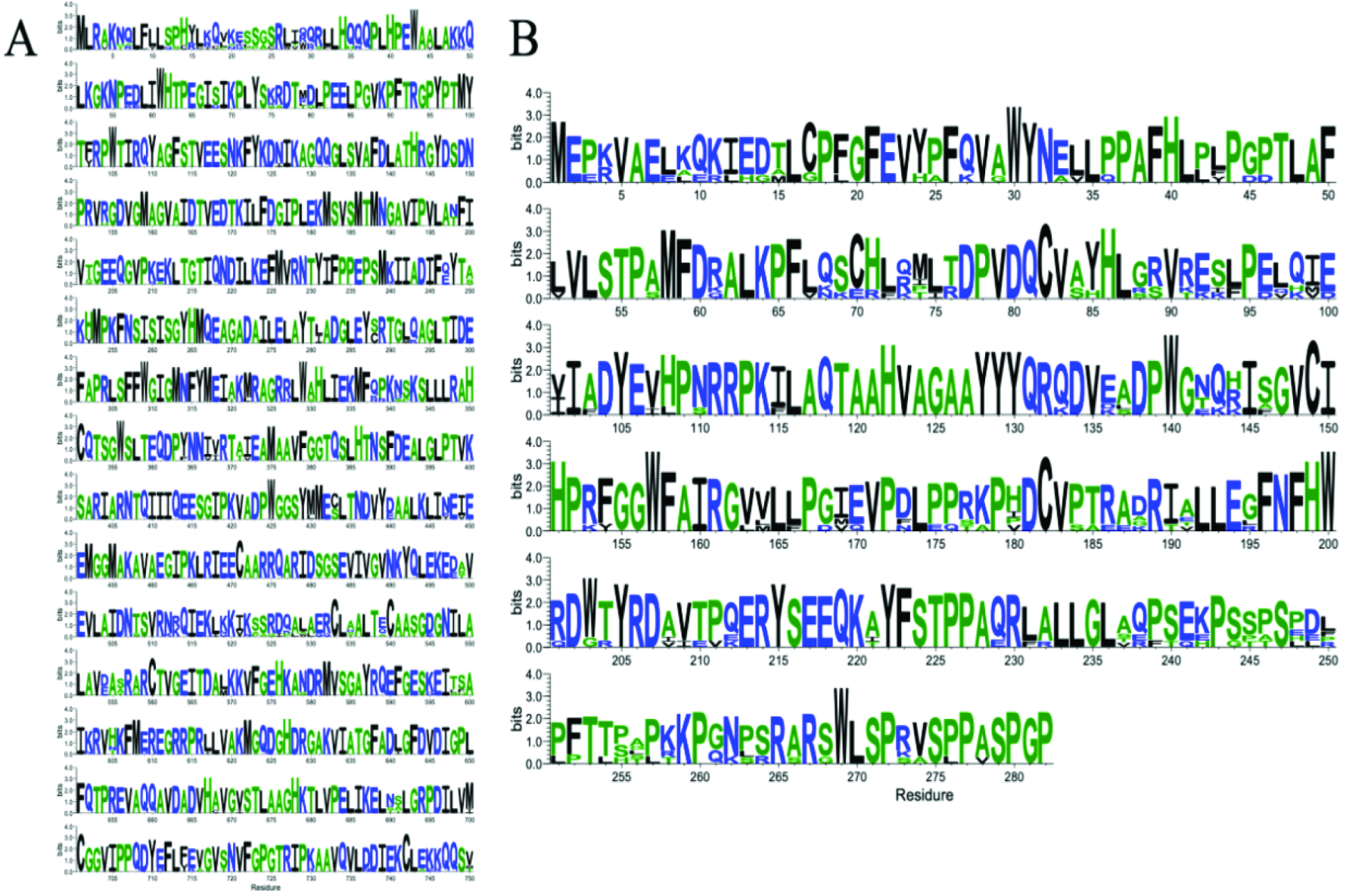


Figure S6. The result of MSA. (A) Visualization of *MMUT* sequence conservation generated using WebLogo. (B) Visualization of *MMACHC* sequence conservation generated using WebLogo.

Table S1. A list of 138 related genes in the study

| **No** | **Gene** | **OMIM** | **Disease** |
| --- | --- | --- | --- |
| 1 | PAH | 612349 | Phenylketonuria |
| 2 | PTS | 612719 | Hyperphenylalaninemia, BH4-Deficent, A |
| 3 | QDPR | 612676 | Hyperphenylalaninemia, BH4-Deficent, A |
| 4 | FAH | 613871 | Tyrosinemia, type I |
| 5 | TAT | 613018 | Tyrosinemia, type II |
| 6 | HPD | 609695 | Yrosinemia, type III |
| 7 | BCKDHA | 608348 | Maple syrup urine disease, type Ia |
| 8 | BCKDHB | 248611 | Maple syrup urine disease, type Ib |
| 9 | DBT | 248610 | Maple syrup urine disease, type II |
| 10 | CPS1 | 608307 | Carbamoylphosphate synthetase I deficiency |
| 11 | OTC | 300461 | Ornithine transcarbamylase deficiency |
| 12 | ASS1 | 603470 | Citrullinemia, type I |
| 13 | NAGS | 608300 | N-acetylglutamate synthase deficiency |
| 14 | SLC25A13 | 603859 | Citrullinemia, type II |
| 15 | ASL | 608310 | Argininosuccinic aciduria |
| 16 | ARG1 | 608313 | Argininemia |
| 17 | OAT | 613349 | Ornithine aminotransferase deficiency |
| 18 | SLC25A15 | 603861 | Hyperornithinemia-hyperammonemia-homocitrullinemia syndrome |
| 19 | CBS | 613381 | Cystathionine β -synthase deficiency type homocysteinemia |
| 20 | MAT1A | 610550 | Hypermethioninemia |
| 21 | PRODH | 606810 | Hyperprolinemia, type I |
| 22 | GLDC | 238300 | Glycine encephalopathy |
| 23 | **MMACHC^*^** | 609831 | Methylmalonic aciduria and homocystinuria, cblC type |
| 24 | **MMUT^*^** | 609058 | Methylmalonic aciduria |
| 25 | **MMAA^*^** | 607481 | Methylmalonic aciduria, cblA type |
| 26 | **MMAB^*^** | 607568 | Methylmalonic aciduria, cblB type |
| 27 | PCCA | 232000 | propionic acidemia |
| 28 | PCCB | 232050 | propionic acidemia |
| 29 | IVD | 607036 | Isovaleric acidemia |
| 30 | GCDH | 608801 | Glutaricaciduria, type I |
| 31 | MCCC1 | 609010 | 3-Methylcrotonyl-CoA carboxylase 1 deficiency |
| 32 | MCCC2 | 609014 | 3-Methylcrotonyl-CoA carboxylase 2 deficiency |
| 33 | AUH | 600529 | 3-methylglutaconic aciduria, type I |
| 34 | HMGCL | 613898 | 3-Hydroxy-3-methylglutaryl coenzyme A lyase deficiency |
| 35 | HLCS | 609018 | Holocarboxylase synthetase deficiency |
| 36 | BTD | 609019 | Biotinidase deficiency |
| 37 | ACAT1 | 607809 | β-Ketothiolase deficiency |
| 38 | ACADSB | 600301 | 2-Methylbutyryl-CoA dehydrogenase deficiency |
| 39 | ACAD8 | 604773 | Isobutyryl-CoA dehydrogenase deficiency |
| 40 | HSD17B10 | 300256 | HSD10 mitochondrial disease |
| 41 | L2HGDH | 609584 | L-2-hydroxyglutaric aciduria |
| 42 | SLC22A5 | 603377 | Primary systemic carnitine deficiency |
| 43 | CPT1A | 600528 | Carnitine palmitoyl transferase IA deficiency |
| 44 | CPT2 | 600650 | Carnitine palmitoyl transferase IIA deficiency |
| 45 | SLC25A20 | 613698 | Carnitine-acylcarnitine translocase deficiency |
| 46 | ACADS | 606885 | Short-chain acyl-coa dehydrogenase deficiency |
| 47 | ACADM | 607008 | Medium-chain acyl-coa dehydrogenase deficiency |
| 48 | ACADVL | 609575 | very long-chain acyl-CoA dehydrogenase deficiency |
| 49 | HADHA | 600890 | Long-chain hydroxyacyl-coa dehydrogenase deficiency, Mitochondrial trifunctional protein deficiency 1 |
| 50 | HADHB | 143450 | Mitochondrial trifunctional protein deficiency 1 |
| 51 | ETFA | 608053 | Glutaricaciduria, type II |
| 52 | ETFB | 130410 | Glutaricaciduria, type II |
| 53 | ETFDH | 231675 | Glutaricaciduria, type II |
| 54 | ETHE1 | 608451 | Ethylmalonic encephalopathy |
| 55 | MLYCD | 606761 | Malonyl-CoA decarboxylase deficiency |
| 56 | SMPD1 | 607608 | Niemann-Pick disease, type A/B |
| 57 | NPC1 | 607623 | Niemann-Pick disease, type C1 |
| 58 | NPC2 | 601015 | Niemann-Pick disease, type C2 |
| 59 | GBA | 606463 | Gaucher Disease |
| 60 | GLA | 300644 | Fabry disease |
| 61 | IDUA | 252800 | Mucopolysaccharidosis Ih; Mucopolysaccharidosis Ih/s; Mucopolysaccharidosis Is |
| 62 | IDS | 300823 | Mucopolysaccharidosis, type II |
| 63 | GALNS | 612222 | Mucopolysaccharidosis, type IVA |
| 64 | GLB1 | 611458 | GM1-gangliosidosis, type I/II/III; Mucopolysaccharidosis, type IVB |
| 65 | ARSB | 611542 | Mucopolysaccharidosis, type VI |
| 66 | G6PC | 613742 | Glycogen storage disease, type Ia |
| 67 | SLC37A4 | 602671 | Glycogen storage disease, type Ib/Ic |
| 68 | GAA | 606800 | Glycogen storage disease,, type II |
| 69 | AGL | 610860 | Glycogen storage disease , type III |
| 70 | CYP11B1 | 610613 | Congenital adrenal hyperplasia due to 11-beta-hydroxylase deficiency |
| 71 | GALC | 606890 | Krabbe disease |
| 72 | PSAP | 176801 | Krabbe disease, atypical;  Metachromatic leukodystrophy due to SAP-b deficiency;  Gaucher disease, atypical;  Combined SAP deficiency |
| 73 | ARSA | 607574 | Metachromatic leukodystrophy |
| 74 | ABCD1 | 300371 | X-linked adrenoleukodystrophy |
| 75 | ATP7B | 606882 | Wilson disease |
| 76 | FGFR3 | 134934 | Achondroplasia |
| 77 | HBB | 141900 | Beta-thalassemia |
| 78 | F9 | 300746 | Hemophilia B |
| 79 | GALT | 606999 | Galactosemia |
| 80 | GJB2 | 121011 | Autosomal recessive deafness 1A |
| 81 | GJB3 | 603324 | Autosomal dominant deafness 2B |
| 82 | SLC26A4 | 605646 | Autosomal recessive deafness type 4 with enlarged vestibular aqueduct, Pendred syndrome |
| 83 | MT-RNR1 | 180450 | Mitochondrial non-syndromic sensorineural hearing loss |
| 84 | ALDOB | 612724 | Hereditary fructose intolerance |
| 85 | DNAJC12 | 606060 | Mild hyperphenylalaninemia, non-BH4-deficient |
| 86 | GCH1 | 600225 | Dopa-Responsive Dystonia; Hyperphenylalaninemia B, BH4-Deficient |
| 87 | PCBD1 | 126090 | Hyperphenylalaninemia, BH4-deficient, D |
| 88 | MTHFR | 607093 | Homocystinuria due to MTHFR deficiency |
| 89 | DLD | 238331 | Dihydrolipoamide dehydrogenase deficiency |
| 90 | PC | 608786 | Pyruvate carboxylase deficiency |
| 91 | HADH | 601609 | 3-hydroxyacyl-CoA dehydrogenase deficiency |
| 92 | NADK2 | 615787 | 2,4-dienoyl-CoA reductase deficiency |
| 93 | **SUCLA2^*^** | 603921 | SUCLA2-related encephalomyopathic mitochondrial DNA depletion syndrome |
| 94 | **SUCLG1^*^** | 611224 | SUCLG1-related encephalomyopathic mitochondrial DNA depletion syndrome |
| 95 | **LMBRD1^*^** | 612625 | Methylmalonic aciduria and homocystinuria, cblF type |
| 96 | **HCFC1^*^** | 300019 | Methylmalonic aciduria and homocysteinemia, cblX type |
| 97 | TAZ | 300394 | Barth syndrome |
| 98 | GNMT | 606628 | Glycine N-methyltransferase deficiency |
| 99 | AHCY | 180960 | Hypermethioninemia |
| 100 | **MMADHC** | 611935 | Methylmalonic aciduria and homocystinuria, cblD type |
| 101 | **MCEE^*^** | 608419 | Methylmalonyl-CoA epimerase deficiency |
| 102 | **ABCD4^*^** | 603214 | Methylmalonic aciduria and homocystinuria, cblJ type |
| 103 | LDLR | 606945 | Familial hypercholesterolemia, type 1 |
| 104 | APOB | 107730 | Familial hypercholesterolemia, type 2 |
| 105 | GALK1 | 604313 | Galactokinase Deficiency |
| 106 | GALE | 606953 | Galactose epimerase deficiency |
| 107 | PCSK9 | 607786 | Familial hypercholesterolemia, type 3 |
| 108 | LDLRAP1 | 605747 | Familial hypercholesterolemia, type 4 |
| 109 | ABCG5 | 605459 | Sitosterolemia, type 1 |
| 110 | ABCG8 | 605460 | Sitosterolemia, type 2 |
| 111 | ATP7A | 300011 | Menkes disease |
| 112 | SGSH | 605270 | Mucopolysaccharidosis, type IIIA |
| 113 | NAGLU | 609701 | Xonal Charcot-Marie-Tooth disease type 2L, Mucopolysaccharidosis, type IIIB |
| 114 | GUSB | 611499 | Mucopolysaccharidosis, type VII |
| 115 | GNPTAB | 607840 | Mucolipidosis II alpha/beta, Mucolipidosis III alpha/beta |
| 116 | SPR | 182125 | DOPA-responsive dystonia due to sepiaterin reductase deficiency |
| 117 | MTR | 156570 | Homocystinuria-megaloblastic anemia, cblG complementation type |
| 118 | MTRR | 602568 | Homocystinuria-megaloblastic anemia, cblE type |
| 119 | **ACSF3^*^** | 614245 | Combined malonic and methylmalonic aciduria |
| 120 | **CD320^*^** | 606475 | Methylmalonic aciduria, transient, due to transcobalamin receptor defect |
| 121 | **ALDH6A1^*^** | 603178 | Methylmalonate semialdehyde dehydrogenase deficiency |
| 122 | HEXA | 606869 | Tay-Sachs disease |
| 123 | PYGL | 613741 | Glycogen storage disease, type VI |
| 124 | PHKA2 | 300798 | Glycogen storage disease, type IX |
| 125 | ALDH7A1 | 107323 | Pyridoxine-dependent epilepsy |
| 126 | SLC2A1 | 138140 | GLUT1 deficiency syndrome |
| 127 | PNPO | 603287 | Pyridoxamine 5'-phosphate oxidase deficiency |
| 128 | PHGDH | 606879 | Neu-Laxova syndrome 1, Phosphoglycerate dehydrogenase deficiency |
| 129 | PSAT1 | 610936 | Phosphoserine aminotransferase deficiency, Neu-Laxova syndrome 2 |
| 130 | PSPH | 172480 | Phosphoserine phosphatase deficiency |
| 131 | GATM | 602360 | Cerebral creatine deficiency syndrome 3 |
| 132 | GAMT | 601240 | Cerebral creatine deficiency syndrome 2 |
| 133 | SLC6A8 | 300036 | Cerebral creatine deficiency syndrome 1 |
| 134 | ADA | 608958 | Adenosine deaminase deficiency, partial, Adenosine deaminase deficiency severe combined immunodeficiency |
| 135 | TH | 191290 | Segawa syndrome |
| 136 | DDC | 107930 | Aromatic L-amino acid decarboxylase deficiency |
| 137 | UGT1A1 | 191740 | Gilbert syndrome, Crigler-Najjar syndrome |
| 138 | SLC10A1 | 182396 | Sodium-taurocholate cotransporting polypeptide deficiency |

**^*^:** 14 MMA-related genes.

Table S2. Evidence of ACMG pathogenicity classification for VUS sites in the study

|  | **Variants** | **ACMG** | **Pathogenicity** |
| --- | --- | --- | --- |
| *MUT* | c.191C>T | PM2_Supporting;PP3_Moderate | VUS |
|  | c.232A>G | PM2_Supporting | VUS |
|  | c.437A>G | PM2_Supporting;PP3_Strong | VUS |
|  | c.1159A>C | PM2_Supporting;PP3_Strong;PM3_Moderate | VUS→LP* |
|  | c.1208G>A | PM2_Supporting;PP3_Strong;PP4 | VUS |
|  | c.1231A>G | PM2_Supporting | VUS |
|  | c.1264G>A | PM2_Supporting;PP3_Moderate | VUS |
|  | c.1286A>G | PP3_Moderate;PM2_Supporting;PP4 | VUS |
|  | c.1631G>A | PM2_Supporting | VUS |
|  | c.1963C>T | PM2_Supporting;PP3 | VUS |
|  | c.2084C>T | PM2_Supporting | VUS |
| *MMACHC* | c.403G>T | PM2_Supporting;PP3 | VUS |
|  | c.426C>A | PM2_Supporting;BP4_Moderate | VUS |
|  | c.565C>T | PM2_Supporting;PP4;PM5 | VUS |
|  | c.569T>G | PM2_Supporting;PP3_Moderate | VUS |
|  | c.580G>A | PM2_Supporting;PP3_Moderate | VUS |
|  | c.641G>A | PM2_Supporting | VUS |
|  | c.683C>T | PM2_Supporting;PM3;PP4 | VUS |
|  | c.754T>C | PM2_Supporting;BP4 | VUS |

***:** According to the ACMG genetic variation classification standard, c. 1159A > C was classified as VUS during the study.Due to the previous literature published by our research group, the current database has been upgraded to likely pathogenic.

Table S3. Structural effects on *MMUT/MMACHC* due to variants obtained from the HOPE Server

| **Protein variants** | **Structure^a^** | **Properties** |
| --- | --- | --- |
| MUT  R655C | 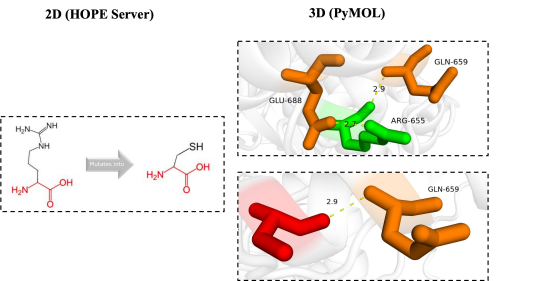 | - This mutation is located in the Vitamin B12 binding domain. - Mutant residue is smaller than the wild-type residue. - Mutant residue is more hydrophobic than the wild-type residue. - Wild-type residue form hydrogen bond with GLN-659, GLU-688. - Mutant residue form hydrogen bond with GLN-659. |
| MUT  Y429C | 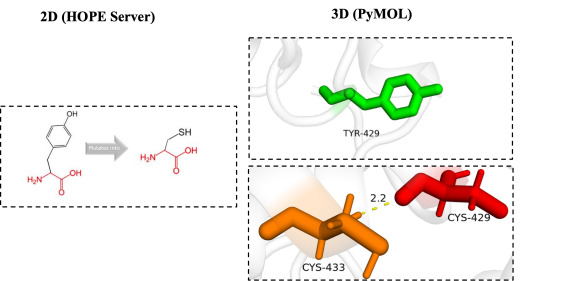 | - This mutation is located near a highly conseverd domain whicn is important for binding of other molecules. - Mutant residue is smaller than the wild-type residue. - Mutant residue is more hydrophobic than the wild-type residue. - Wild-type residue didn’t form hydrogen bond with other residues. - Mutant residue form hydrogen bond with CYS-433. |
| MUT  A555T | 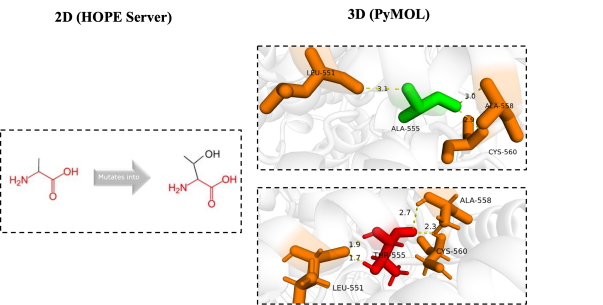 | - This mutation is located near a highly conseverd domain whicn is important for binding of other molecules.. - Mutant residue is bigger than the wild-type residue. - Mutant residue is less hydrophobic than the wild-type residue. - Wild-type residue form hydrogen bond with LEU-551, ALA-558,CYS-560. - Mutant residue form hydrogen bond with LEU-551, ALA-558, CYS-560. |
| MUT  Y146C | 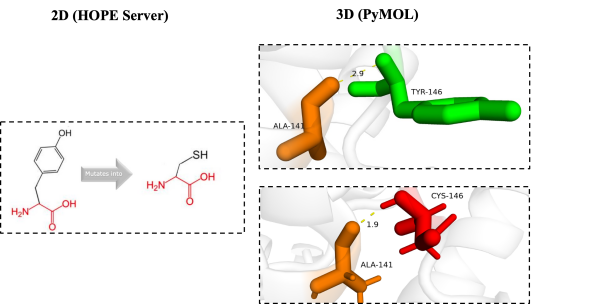 | - This mutation is located near a highly conseverd domain whicn is important for binding of other molecules. - Mutant residue is smaller than the wild-type residue. - Mutant residue is more hydrophobic than the wild-type residue. - Wild-type residue form hydrogen bond with ALA-141. - Mutant residue form hydrogen bond with ALA-141. |
| MUT  P695L | 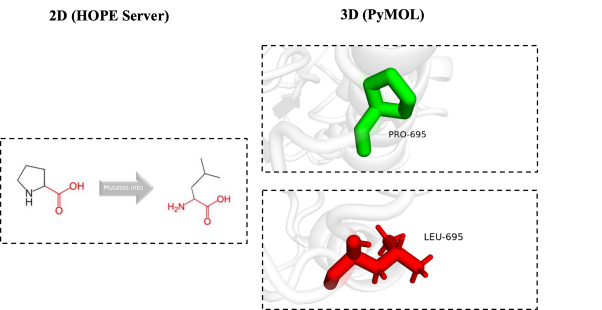 | - This mutation is located in the Vitamin B12 binding domain. - Mutant residue is bigger than the wild-type residue. - Mutation can disturb the special conformation of proline. - Wild-type residue didn’t form hydrogen bond with other residues. - Mutant residue didn’t form hydrogen bond with other residues. |
| MUT  A422T | 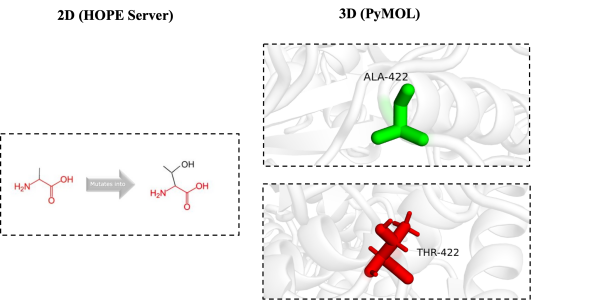 | - This mutation is located near a highly conseverd domain. - Mutant residue is bigger than the wild-type residue. - Mutant residue is more hydrophobic than the wild-type residue. - Wild-type residue didn’t form hydrogen bond with other residues. - Mutant residue didn’t form hydrogen bond with other residues. |
| MUT  T78A | 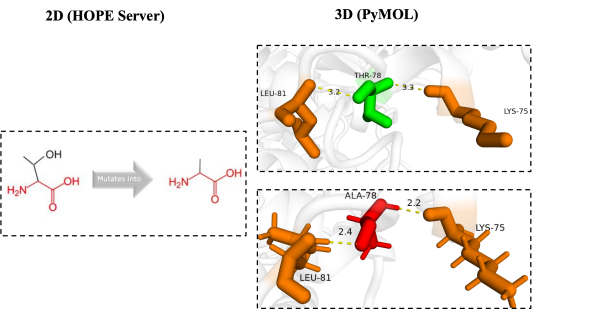 | - This mutation is located near a highly conseverd domain. - Mutant residue is smaller than the wild-type residue. - Mutant residue is more hydrophobic than the wild-type residue. - Wild-type residue form hydrogen bond with LYS-75, LEU-81. - Mutant residue form hydrogen bond with LYS-75, LEU-81. |
| MUT  R108H | 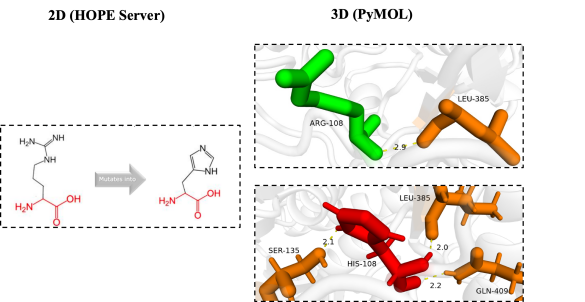 | - This mutation is located within a stetch of residues of annotated in UniProt as a special region:malonyl-CoA binding. - Mutant residue is smaller than the wild-type residue. - Wild-type residue form hydrogen bond with LEU-385. - Mutant residue form hydrogen bond with SER-135,LEU-385,GLN-409. |
| MUT  T387P | 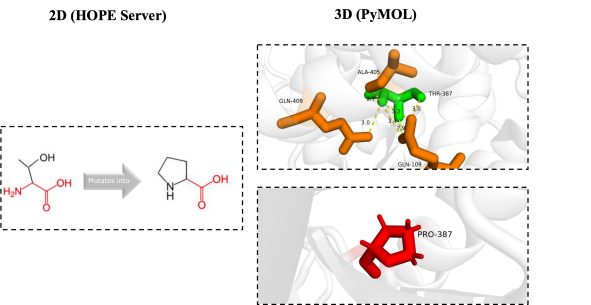 | - This mutation is located near a highly conseverd domain whicn is important for binding of other molecules. - Mutant residue is more hydrophobic than the wild-type residue. - Wild-type residue form hydrogen bond with GLN -109, ALA-405,GLN-409. - Mutant residue didn’t form hydrogen bond with other residues. |
| MUT  I411V | 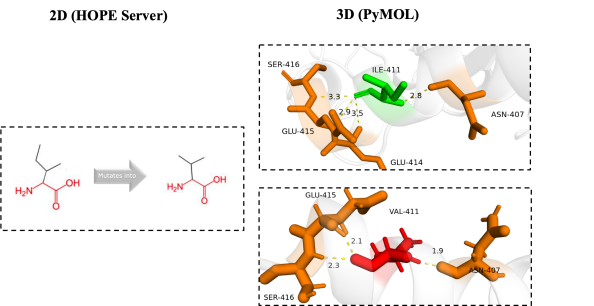 | - This mutation is located near a highly conseverd domain whicn is important for binding of other molecules. - Mutant residue is smaller than the wild-type residue. - Wild-type residue form hydrogen bond with ASN-407,GLU-414,GLU-415,SER-416. - Mutant residue form hydrogen bond with ASN-407,GLU 415,SER-416. |
| MUT  R403Q | 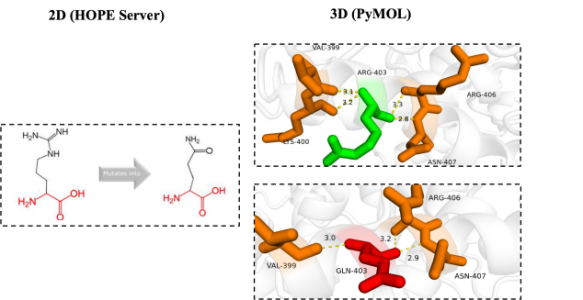 | - This mutation is located near a highly conseverd domain whicn is important for binding of other molecules. - Mutant residue is smaller than the wild-type residue. - Wild-type residue form hydrogen bond with VAL-399,LYS-400,ARG-406,ASN-407. - Mutant residue form hydrogen bond withVAL-399,ARG-406,ASN-407. |
| MUT  G544E | 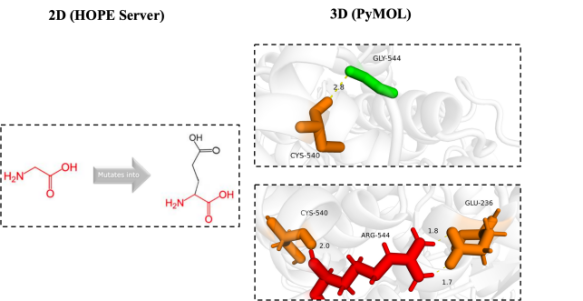 | - This mutation is located near a highly conseverd domain whicn is important for binding of other molecules. - Mutant residue is bigger than the wild-type residue. - Mutant charge is negative, wild-type charge was neutral. - Mutant residue is more hydrophobic than the wild-type residue. - Wild-type residue form hydrogen bond with CYS-540. - Mutant residue form hydrogen bond with GLU-236,CYS-540. |
| MUT  P64L | 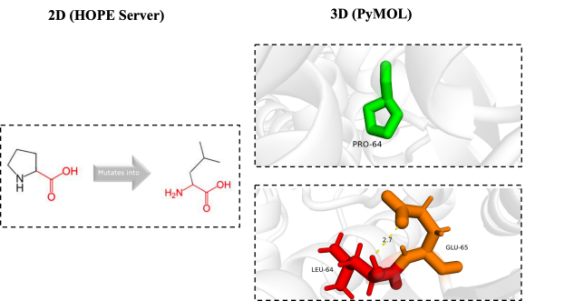 | - This mutation is located near a highly conseverd domain whicn is important for binding of other molecules. - Mutation can disturb the special conformation of proline. - Mutant residue is bigger than the wild-type residue. - Wild-type residue didn’t form hydrogen bond with other residues. - Mutant residue form hydrogen bond with GLU-65. |
| MUT  G427D | 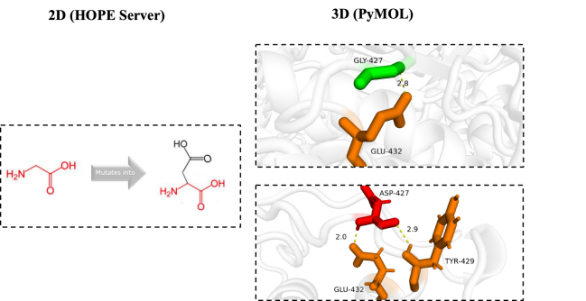 | - This mutation is located near a highly conseverd domain whicn is important for binding of other molecules. - Mutant residue is bigger than the wild-type residue. - Mutant residue charge is negative,wild-type residue charge was neutral. - Mutant residue is more hydrophobic than the wild-type residue. - Wild-type residue form hydrogen bond with GLU-432. - Mutant residue form hydrogen bond with TYR-429,GLU-432 |
| MMACHC  A228V | 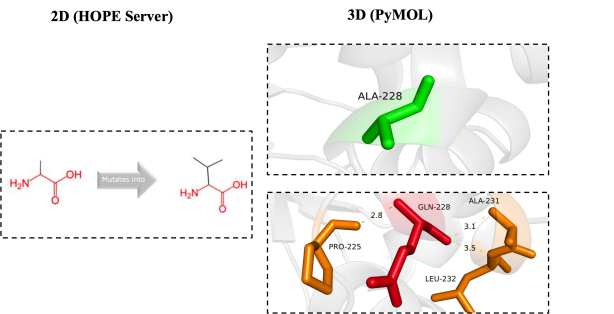 | - This mutation is located near a highly conseverd domain whicn is important for binding of other molecules. - Mutant residure in the 3D-structure can be seen that the wild-type residue is located in an α-helix. - Mutant residue is bigger than the wild-type residue. - Wild-type residue didn’t form hydrogen bond with other residues. - Mutant residue form hydrogen bond with PRO-225,ALA-231,LEU-232. |
| MMACHC  V135L | 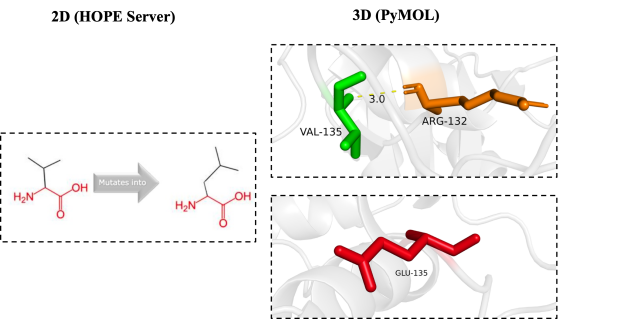 | - This mutation is located near a highly conseverd domain whicn is important for binding of other molecules. - Mutant residue prefers to be in another secondary structure. - Mutant residue is bigger than the wild-type residue. - Wild-type residue form hydrogen bond with ARG-132. - Mutant residue didn’t form hydrogen bond with other residues. |
| MMACHC  R161Q | 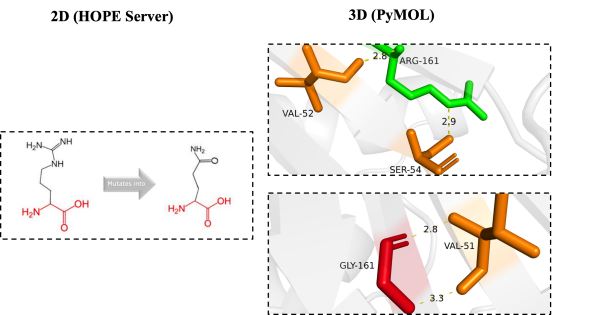 | - This mutation is located near a highly conseverd domain whicn is important for binding of other molecules. - Mutant residue is smaller than the wild-type residue. - Mutant residue charge is neutral,wild-type residue charge was positive. - Wild-type residue form hydrogen bond with VAL-52,SER-54. - Mutant residue form hydrogen bond withVAL-51. |
| MMACHC  I190S | 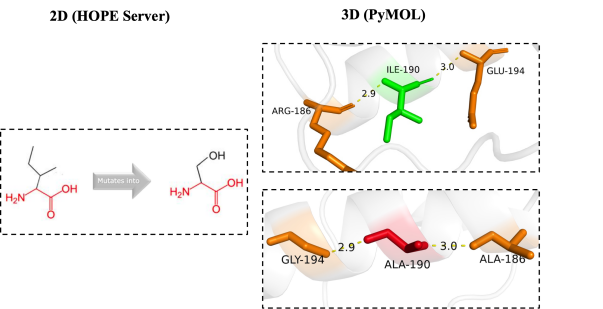 | - This mutation is located near a highly conseverd domain whicn is important for binding of other molecules. - Mutant residue is smaller than the wild-type residue. - Mutant residue is more hydrophobic than the wild-type residue. - Wild-type residue form hydrogen bond with ARG-186,GLU-194. - Mutant residue form hydrogen bond with ALA-186,GLY-194 |
| MMACHC  E194K | 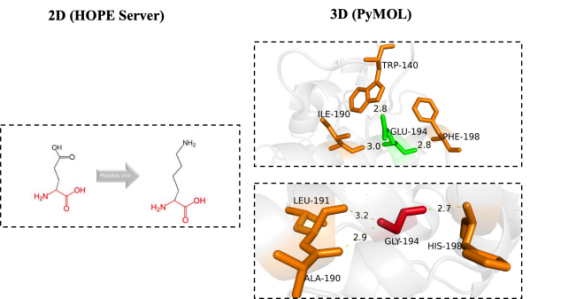 | - This mutation is located near a highly conseverd domain whicn is important for binding of other molecules. - Mutant residue is bigger than the wild-type residue. - Mutant residue charge is positive,wild-type charge was negative. - Wild-type residue form hydrogen bond with TRP-140,ILE-190,PHE-198. - Mutant residue form hydrogen bond with ALA-190,LEU-191,HIS-198. |
| MMACHC  R189C | 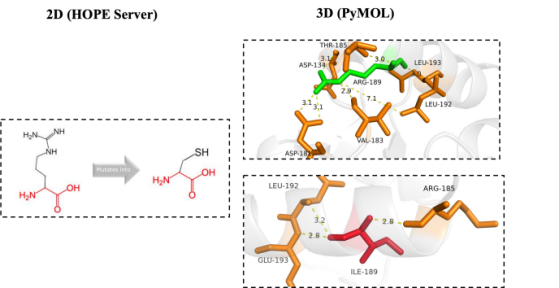 | - This mutation is located near a highly conseverd domain whicn is important for binding of other molecules. - Mutant residue is smaller than the wild-type residue. - Mutant residue charge is neutral,wild-type residue charge was positive. - Mutant residue is more hydrophobic than the wild-type residue. - Wild-type residue form hydrogen bond with ASP-134,ASP-181,VAL-183,THR-185,LEU-192,LEU-193. - Mutant residue form hydrogen bond with ARG-185,LEU-192,GLU-193. |
| MMACHC  R214H | 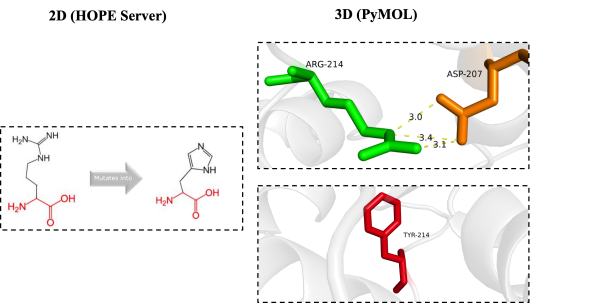 | - This mutation is located near a highly conseverd domain whicn is important for binding of other molecules. - Mutant residue is smaller than the wild-type residue. - Mutant residue charge is neutral,wild-type charge was positive - Wild-type residue form hydrogen bond with ASP-207. Mutant residue didn’t form hydrogen bond with other residues. |
| MMACHC  N142K | 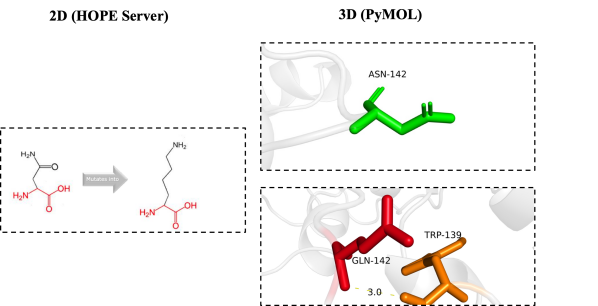 | - This mutation is located near a highly conseverd domain whicn is important for binding of other molecules. - Mutant residue is bigger than the wild-type residue. - Mutant residue charge is positive,wild-type residue charge was neutral. - Wild-type residue didn’t form hydrogen bond with other residues. Mutant residue form hydrogen bond with TPR-139. |

^a^ Green is the wild-type residue;Red is the mutation residue;Orange is the interaction residus.

Table S4. Differences in the binding pockets of the wild-type protein and mutant proteins identified using the CASTp 3.0 server

| **Position** | **Different in Binding Pocket** | | **Wild-type** | | **Variants** | |
| --- | --- | --- | --- | --- | --- | --- |
|  | **Wild-type** | **variant** | **Area** | **Volume** | **Area** | **Volume** |
| MUT  R655C | NA | 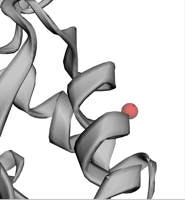 | NA | NA | 0.814 | 0.03 |
| MUT  Y429C | NA | NA | NA | NA | 18.226 | 12.249 |
| MUT  A555T | NA | 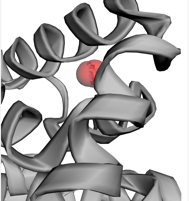 | NA | NA | 3.858 | 0.632 |
| MUT  Y146C | 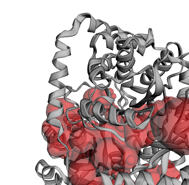 | 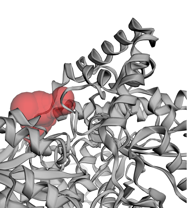 | 4114.07 | 3523.469 | 206.136 | 135.558 |
| MUT  P695L | 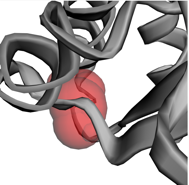 | 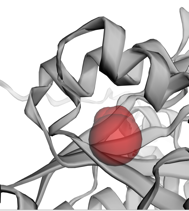 | 39.73 | 15.156 | 25.548 | 10.264 |
| MUT  A422T | 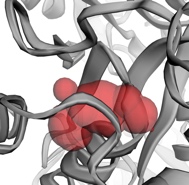 | 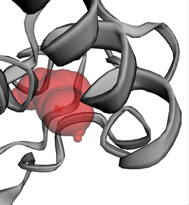 | 55.521 | 11.942 | 68.049 | 17.978 |
| MUT  T78A | 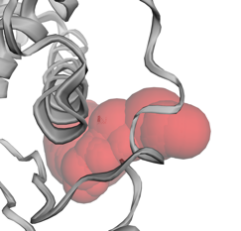 | 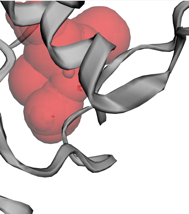 | 217.35 | 188.722 | 234.998 | 168.862 |
| MUT  R108H | 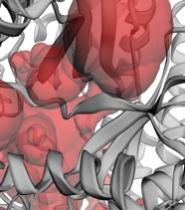 | 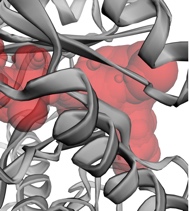 | 4114.07 | 3523.469 | 1678.951 | 992.463 |
| MUT  T387P | NA | NA | NA | NA | NA | NA |
| MUT  I411V | NA | NA | NA | NA | NA | NA |
| MUT  R403Q | NA | 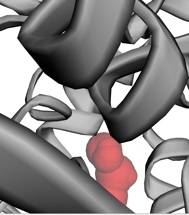 | NA | NA | 26.934 | 5.921 |
| MUT  G544E | NA | 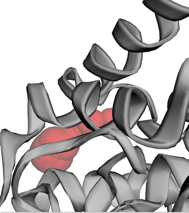 | 17.306 | 3.022 | 28.625 | 6.807 |
| MUT  P64L | NA | 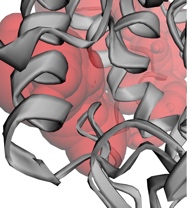 | NA | NA | 1362.947 | 1369.697 |
| MUT  G427D | NA | 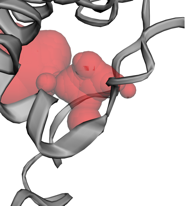 | NA | NA | 155.385 | 88.229 |
| MMACHC  A228V | 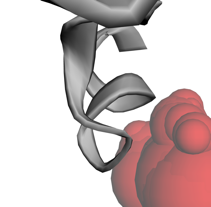 | 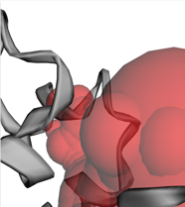 | 114.102 | 76.652 | 677.902 | 929.782 |
| MMACHC  V135L | 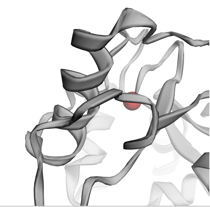 | 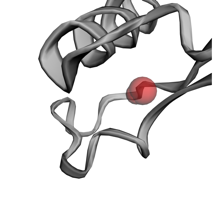 | 1.391 | 0.07 | 2.672 | 0.237 |
| MMACHC  R161Q | 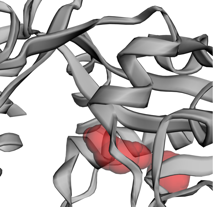 | 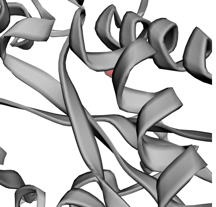 | 61.579 | 17.698 | 0.955 | 0.029 |
| MMACHC  I190S | 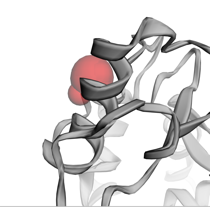 | 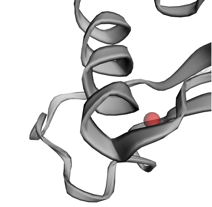 | 17.592 | 6.35 | 0.614 | 0.021 |
| MMACHC  E194K | 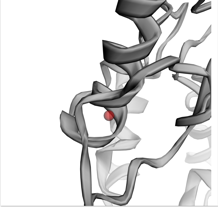 | 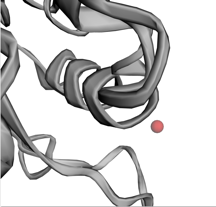 | 0.167 | 0.003 | 0.103 | 0.004 |
| MMACHC  R189C | 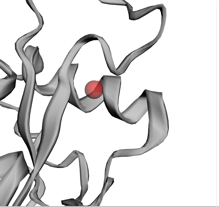 | 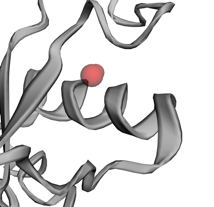 | 1.391 | 0.07 | 1.524 | 0.09 |
| MMACHC  R214H | 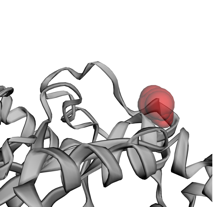 | 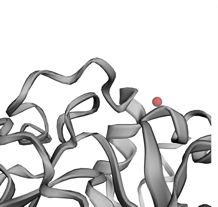 | 30.104 | 11.285 | 0.293 | 0.007 |
| MMACHC  N142K | NA | 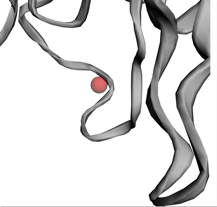 | NA | NA | 0.492 | 0.025 |

Table S5: Predictions for all missense nsSNPs by seven tools with their respective scores

| **Gene** | **Position** | **PANTHER** | | **PolyPhen-2** | | **SIFT** | | **FATHMM** | | **DeepDDG** |
| --- | --- | --- | --- | --- | --- | --- | --- | --- | --- | --- |
|  |  | **Time** | **Messange** | **Prediction** | **Prediction** | **Tolerance Index** | **Message** | **Prediction** | **SCORE** | **Prediction** |
| *MUT* | R655C | 455 | probably damaging | PROBABLY DAMAGING | disease | 0.05 | predicted to AFFECT PROTEIN FUNCTION | DAMAGING | -3.98 | Decrease Stability |
| *MUT* | Y429C | 4200 | probably damaging | PROBABLY DAMAGING | disease | 0.07 | predicted to be TOLERATED | DAMAGING | -5.32 | Decrease Stability |
| *MUT* | A555T | 4200 | probably damaging | PROBABLY DAMAGING | neutral | 0 | predicted to AFFECT PROTEIN FUNCTION | DAMAGING | -6.12 | Decrease Stability |
| *MUT* | Y146C | 4200 | probably damaging | PROBABLY DAMAGING | disease | 0.01 | predicted to AFFECT PROTEIN FUNCTION | DAMAGING | -5.04 | Decrease Stability |
| *MUT* | P695L | 750 | probably damaging | BENIGN | neutral | 0.04 | predicted to AFFECT PROTEIN FUNCTION | DAMAGING | -4.26 | Decrease Stability |
| *MUT* | A422T | 750 | probably damaging | PROBABLY DAMAGING | neutral | 0.46 | predicted to be TOLERATED | DAMAGING | -4.9 | Decrease Stability |
| *MUT* | T78A | 1500 | probably damaging | BENIGN | neutral | 1 | predicted to be TOLERATED | DAMAGING | -4.77 | Decrease Stability |
| *MUT* | R108H | 4200 | probably damaging | PROBABLY DAMAGING | disease | 0 | predicted to AFFECT PROTEIN FUNCTION | DAMAGING | -5.4 | Decrease Stability |
| *MUT* | T387P | 4200 | probably damaging | PROBABLY DAMAGING | disease | 0.07 | predicted to be TOLERATED | DAMAGING | -5.17 | Decrease Stability |
| *MUT* | I411V | 4200 | probably damaging | BENIGN | neutral | 0.52 | predicted to be TOLERATED | DAMAGING | -3.98 | Decrease Stability |
| *MUT* | R403Q | 4200 | probably damaging | PROBABLY DAMAGING | neutral | 0.17 | predicted to AFFECT PROTEIN FUNCTION | DAMAGING | -5.32 | Decrease Stability |
| *MUT* | G544E | 1237 | probably damaging | BENIGN | disease | 0.29 | predicted to be TOLERATED | DAMAGING | -6.12 | Decrease Stability |
| *MUT* | P64L | 4200 | probably damaging | BENIGN | neutral | 0.59 | predicted to AFFECT PROTEIN FUNCTION | DAMAGING | -5.32 | Decrease Stability |
| *MUT* | G427D | 4200 | probably damaging | PROBABLY DAMAGING | disease | 0 | predicted to AFFECT PROTEIN FUNCTION | DAMAGING | -6.12 | Decrease Stability |
| *MMACHC* | A228V | 455 | probably damaging | BENIGH | neutral | 0.24 | predicted to be TOLERATED | DAMAGING | -3.79 | Decrease Stability |
| *MMACHC* | V135L | 455 | probably damaging | PROBABLY DAMAGING | neutral | 0.68 | predicted to be TOLERATED | DAMAGING | -4.74 | Decrease Stability |
| *MMACHC* | R161Q | 1500 | probably damaging | PROBABLY DAMAGING | disease | 0 | predicted to AFFECT PROTEIN FUNCTION | DAMAGING | -4.39 | Decrease Stability |
| *MMACHC* | I190S | 1500 | probably damaging | PROBABLY DAMAGING | disease | 0.05 | predicted to AFFECT PROTEIN FUNCTION | DAMAGING | -4.33 | Decrease Stability |
| *MMACHC* | E194K | 750 | probably damaging | PROBABLY DAMAGING | disease | 0 | predicted to AFFECT PROTEIN FUNCTION | DAMAGING | -4.57 | Decrease Stability |
| *MMACHC* | R189C | 1500 | probably damaging | PROBABLY DAMAGING | disease | 0 | predicted to AFFECT PROTEIN FUNCTION | DAMAGING | -4.32 | Decrease Stability |
| *MMACHC* | R214H | 176 | probably benign | PROBABLY DAMAGING | neutral | 0.03 | predicted to AFFECT PROTEIN FUNCTION | DAMAGING | -3.59 | Decrease Stability |
| *MMACHC* | N142K | 91 | probably benign | BENIGH | neutral | 1 | predicted to be TOLERATED | DAMAGING | -4.22 | Decrease Stability |

Table S6. Conservation analysis results predicted by Consurf for *MMACHC* variant sites

| **Gene** | **Variant sites** | **Position** | **Score** |
| --- | --- | --- | --- |
| *MMACHC* | c.403G>T | V135L | 7 |
| *MMACHC* | c.426C>A | N142K | 3 |
| *MMACHC* | c.482G>A | R161Q | 9 |
| *MMACHC* | c.565C>T | R189C | 6 |
| *MMACHC* | c.569T>G | I190S | 6 |
| *MMACHC* | c.580G>A | E194K | 6 |
| *MMACHC* | c.641G>A | R214H | 6 |
| *MMACHC* | c.683C>T | A228V | 5 |

Table S7. Docking result of *MMACHC* protein with cob(II)alamin

| **Variant sites** | **Position** | **Ligand** | **Wild-type binding**  **affinity (kcal/mol)** | **Mutation binding**  **affinity (kcal/mol)** |
| --- | --- | --- | --- | --- |
| *c.403G>T* | V135L | cob(II)alamin | -4.50 | -4.40 |
| *c.426C>A* | N142K | cob(II)alamin | -4.82 | -4.78 |
| *c.482G>A* | R161Q | cob(II)alamin | -8.68 | -1.77 |
| *c.565C>T* | R189C | cob(II)alamin | -5.69 | -5.55 |
| *c.569T>G* | I190S | cob(II)alamin | -5.61 | -5.35 |
| *c.580G>A* | E194K | cob(II)alamin | -5.67 | -4.49 |
| *c.641G>A* | R214H | cob(II)alamin | -4.96 | -4.24 |
| *c.683C>T* | A228V | cob(II)alamin | -5.90 | -3.80 |

Table S8. Number of newborns with carrying variants in *MMUT* and *MMACHC* in the study

| Gene | Variants | Number |
| --- | --- | --- |
| *MMACHC* | c.403G>T | 41 |
|  | c.426C>A | 9 |
|  | c.565C>T | 11 |
|  | c.569T>G | 21 |
|  | c.580G>A | 12 |
|  | c.641G>A | 11 |
|  | c.683C>T | 100 |
|  | c.754T>C | 14 |
|  | c.398_399delAA | 22 |
|  | c.482G>A | 40 |
|  | c.567dupT | 21 |
|  | c.609G>A | 76 |
|  | c.658_660delAAG | 66 |
| *MUT* | c.1159A>C | 16 |
|  | c.1208G>A | 15 |
|  | c.1231A>G | 16 |
|  | c.1264G>A | 16 |
|  | c.1286A>G | 147 |
|  | c.1631G>A | 9 |
|  | c.191C>T | 8 |
|  | c.1963C>T | 152 |
|  | c.2084C>T | 22 |
|  | c.232A>G | 16 |
|  | c.437A>G | 23 |
|  | c.1280G>A | 10 |
|  | c.1630G>T | 15 |
|  | c.1663G>A | 40 |
|  | c.323G>A | 18 |
